# d*Sa*Cas9 enables enhanced transcriptional activation in *Nicotiana benthamiana* compared to its d*Sp*Cas9 ortholog

**DOI:** 10.1186/s12870-026-09174-6

**Published:** 2026-06-04

**Authors:** Aaron M. Klepper, Amir Akhgari, Heiko Rischer

**Affiliations:** VTT Technical Research Centre of Finland Ltd, Espoo, Finland

**Keywords:** d*Sa*Cas9, d*Sp*Cas9, CRISPRa, Transcription regulation, Target gene activation, Transient expression, Dual luciferase

## Abstract

The *Sa*Cas9 from *Staphylococcus aureus* has been shown to be more effective than the commonly used *Sp*Cas9 from *Streptococcus pyogenes* for the generation of mutations in plants and has boosted CRISPR/Cas systems. CRISPRa/Cas systems on the other hand have been focused on the use of *Sp*Cas9. Building on foundational work, we developed a modular CRISPRa/Cas ortholog system based on a single plasmid infiltration for the use and comparison of both *Sa*Cas9 and *Sp*Cas9 for enhanced gene expression. Utilizing the multi-kingdom Golden Gate cloning platform, we integrated various elements to create a widely adaptable system.

For the first time in the current study, we demonstrate that the d*Sa*Cas9 effector induces stronger activation of reporter gene expression by targeted promoters than d*Sp*Cas9 in plants. As both effectors are sharing a common target site for the tested promoter, they could be directly compared with each other showing that the d*Sa*Cas9-effector exhibited a stronger effect in the expression of the reporter genes. This allows fine-tuning the expression of genes by using different effectors. In addition, we could show on the example of the *pNOS* promoter and a truncated version of the *pNOS* promoter, that a lower basic expression can lead to an increased relative induction.

**Supplementary Information:**

The online version contains supplementary material available at 10.1186/s12870-026-09174-6.

## Introduction

In recent years, the exploration of various Cas9 and Cas12 orthologs from different bacteria has gained momentum following the innovative development of the CRISPR/*Sp*Cas9 system based on *Streptococcus pyogenes* Cas9. While the *Sp*Cas9 remains the most adapted used variant, the identification and characterization of alternative Cas9 orthologs have expanded the CRISPR systems, offering distinct advantages in size, protospacer adjacent motif (PAM) specificity, and delivery compatibility. Among these, *Sa*Cas9 from *Staphylococcus aureus* has emerged as a promising candidate in plant biotechnology due to its compact size and efficient nuclear localization [1, 2].

Beyond genome editing, catalytically inactive Cas9 (dCas9) variants have been repurposed for transcriptional regulation through CRISPR activation (CRISPRa) [3, 4] and interference (CRISPRi) [3, 5, 6]. CRISPRa systems enable programmable upregulation of endogenous genes without altering the DNA sequence [3], providing powerful tools for functional genomics, metabolic engineering, and synthetic biology. These systems typically employ dCas9 fused to transcriptional activators and can be further enhanced by RNA scaffolds that recruit additional effectors.

**Fig. 1:**
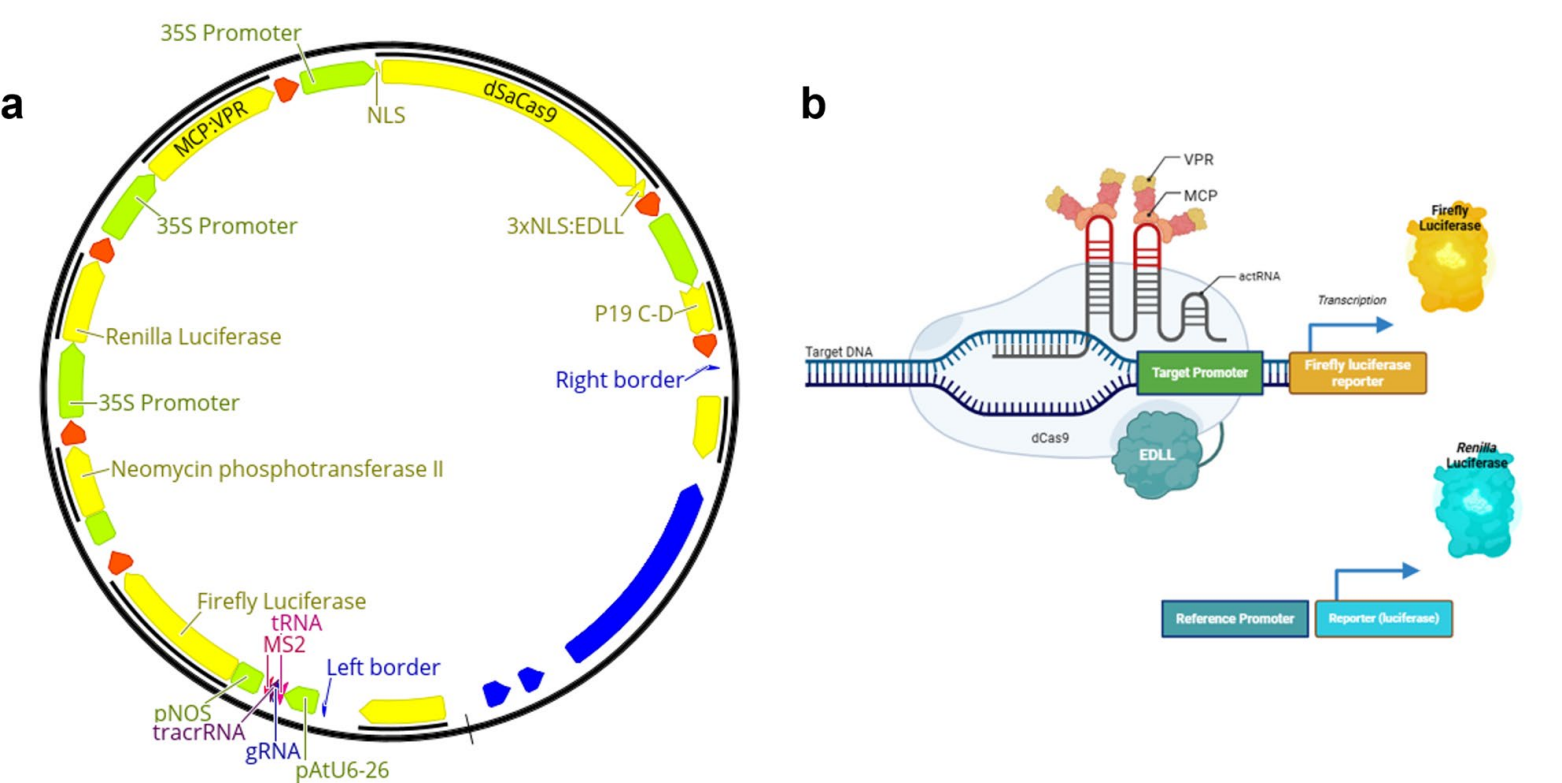
Schematic representation of the modular CRISPRa system used in this study. **a** A single plasmid harbouring all elements of the CRISPRa system is cloned. Coming from the left border, the plasmid is carying the actRNA expression cassette, the firefly luciferase under the pNOS promotor, a resistance marker, the *Renilla* luciferase driven by p35S promoter, the MCP:VPR, the dSaCas9:EDLL and finally a p19 enhancer. Promoters are shown in light green, coding regions in yellow and terminators in orange. **b** A nuclease‑dead Cas9 ortholog (dCas9) is targeted to the pNOS promoter upstream of a firefly luciferase reporter gene via an activating single guide RNA (actRNA). The actRNA is engineered to contain MS2 RNA hairpins, enabling recruitment of MCP:VPR activation domains in trans. In parallel, the dCas9 protein is fused to the plant‑specific EDLL activation domain, forming a dual‑effector CRISPRa configuration. Activation of the target promoter drives firefly luciferase expression, which is quantified relative to a constitutively expressed *Renilla* luciferase reporter driven by the reference p35S promoter. The scheme illustrates the modular architecture of the system, including binding of dCas9 and MCP to the actRNA-DNA complex, enabling direct comparison of different dCas9 orthologs within an otherwise identical CRISPRa framework

Initial enhancements to CRISPRa systems were demonstrated by Chavez et al. [7], who showed that the fusion of multiple transcriptional activators to dCas9 significantly increases gene activation efficiency. Konermann et al. [8] further advanced the field by developing the synergistic activation mediator (SAM) system, which combines a dCas9-VP64 fusion with MS2 RNA aptamer-binding activators p65 and HSF1 to achieve robust transcriptional activation. Building on these advancements, Selma et al. [4, 9] introduced a CRISPRa system with incorporating an EDLL domain fused to dCas9 and MS2 coat protein (MCP) fused to VP64 and p65 (Fig. 1). Remarkably, they identified a spontaneous mutation in the MS2 scaffold that enhanced activation efficiency.

In this study, we adapted the mutation in the MS2 scaffold by Selma et al. [4, 9] into the multi-kingdom Golden Gate cloning platform [10, 11]. Using elements from the works of Binder et al. [10], Chiasson et al. [11], and Klepper [12], we designed a flexible system that supports rapid assembly and adaptation for diverse CRISPR-based applications. While the current study focuses on CRISPRa using dCas9:EDLL and MCP:VPR effectors, the architecture of the system is compatible with modules for CRISPR interference (CRISPRi), such as BRD or SRDX repressors [6] or TEN which has shown to inhibit expression [13], as well as for precision editing technologies. These include base editors using cytidine or adenine deaminases [14, 15] and prime editing systems incorporating reverse transcriptase domains [16–18]. This modularity enables the system to serve as a foundation for expanding programmable gene regulation and editing strategies in plant systems. Furthermore, the Multi-Kingdom Golden-Gate system offers the cloning of a single plasmid that contains all necessary components for CRISPRa analysis [10, 11]. The unified construct simplifies experimental setup, and reduces variability associated with co-infiltration strategies, making it particularly well-suited for transient expression assays in *N. benthamiana*.

Based on previous reports demonstrating high mutation efficiency of *Sa*Cas9 in plant systems [1, 19] and enhanced gene activation in mammalian models [20], we sought to evaluate *Sa*Cas9-based transcriptional activation of a heterologous firefly luciferase reporter driven by the *pNOS* promoter in a transient plant expression system. Subsequently the system was used for direct comparison of both *Sa*Cas9- and well known *Sp*Cas9-based activation. We adapted and integrated key elements from previous studies, including the dual-effector system of dCas9:EDLL and MCP:VPR, and a spontaneously enhanced MS2 scaffold [4, 9], to optimize activation efficiency. We demonstrate that d*Sa*Cas9 consistently induces stronger promoter activation than d*Sp*Cas9 in *N. benthamiana* transient expression. Notably, both effectors share one common target site, while exhibiting several unique sites for each system. The enhanced activation observed with d*Sa*Cas9 at the shared site indicates that the difference is not simply a consequence of position effects. These findings establish d*Sa*Cas9 as a potent alternative for CRISPRa in plants, highlight the utility of our system for gene expression and expand the CRISPRa landscape in plant biotechnology.

## Materials and methods

### Cloning and transformation

All elements were cloned into the multi-kingdom Golden Gate cloning platform following the protocols of Binder et al. [10] and Chiasson et al. [11] with adaptations. For the cut/ligation, we used Thermo Fisher Scientific FastDigest restriction enzymes (*Bpi*I and *Eco*31I/*Bsa*I) and ligase with the ligase buffer, as the FastDigest restriction enzymes are active in the ligase buffer. We decreased the digestion time to 1 min and the ligation time to 5 min, with ten cycles of alternation between digestion and ligation. For cloning the actRNAs, we developed new entry vectors harbouring the tRNA, a ccdB cassette, tracrRNA of *Sp*Cas9 [21] (Addgene plasmid # 80585) or *Sa*Cas9 [1], and the MS2 scRNA [4] (Addgene plasmid # 160593). The ccdB cassette is replaced by the gRNA via a *Bpi*I cut/ligation. Supplementary Table S1 lists the generalised pU6::actRNA architecture for both *Sp*Cas9- and *Sa*Cas9-based systems, in which the spacer is defined as a 20-nt degenerate (N) spacer. The actual actRNA sequences used in this study were generated by substituting this spacer with the individual guide sequences provided in Supplementary Table S2. For multiplexing, different entry vectors with fitting overhangs to each other were used for the LII cloning by a *Bsa*I cut/ligation. In LII, the p*At*U6-26 promoter is combined with a single actRNA or a multiplexing of two or three actRNAs. Final constructs harbour specific actRNAs under the p*At*U6-26 promoter, the firefly luciferase driven by target promoter *pNOS* [10] (Addgene plasmid # 54456) or truncated *pNOS* (*pNOS*_*trc*_) [22] (Addgene plasmid # 68218), a *p35S* driven *Renilla* luciferase, also *p35S* driven MCP:VPR and either d*Sp*Cas9:EDLL or d*Sa*Cas9:EDLL as well as a *p35S* driven RNA silencing suppressor p19. Complete sequence information for all genetic elements, including promoters, coding sequences, and actRNAs, is presented in Supplementary Table S1. Control constructs were designed to incorporate all elements present in the experimental constructs, with the exception of actRNAs. All constructs were cloned into chemically competent Top10 *Escherichia coli*. After DNA purification, constructs were confirmed by restriction enzyme digestion and LI constructs by sequencing. Final plasmids were transformed into electrocompetent *Agrobacterium tumefaciens* EHA105 for tobacco leaf infiltration.

### actRNA design

To design the actRNAs, we utilized the CHOPCHOP web tool (https://chopchop.cbu.uib.no/), a platform specifically optimized for CIRSPR-based applications for a variety of organisms. We uploaded the sequence of our target *pNOS* promoter [10] (Addgene plasmid #54456), which is 83 bp and 44 bp elongated on the 5’- and 3’-end, respectively, compared to the *pNOS*_trc_ [22] (Addgene plasmid # 68218), to the CHOPCHOP webpage and initiated the gRNA design process for either *Sa*Cas9 (NNGRRT PAM) or *Sp*Cas9 (NGG PAM) (Suppl. Figure 1). The platform provided a ranking of potential gRNAs based on their efficiency and off-target effects. We selected our gRNAs according to this ranking, ensuring they were distributed on both the +strand and the -strand.

For the *Sp*Cas9 system, we utilized the PAM sequence NGG, whereas for the *Sa*Cas9 system, the PAM sequence NNGRRT was employed. The gRNAs were then synthesized as two complementary oligonucleotides with each one having a 4-nucleotide overhang to the cloning system.

### Tobacco leaves infiltration

*Agrobacterium* suspensions were infiltrated into the leaves of 6-week-old *N. benthamiana* plants, following the protocol described by Häkkinen et al. [23] with slight modification. Briefly, freshly transformed *A. tumefaciens* strain EHA105 was cultured overnight at 28 °C in liquid LB medium. The bacterial suspension was then collected by centrifugation and resuspended in infiltration buffer containing 10 mM MgSO₄, 200 µM acetosyringone and 10 mM MES (pH 5.6), followed by incubation at room temperature with gentle shaking for 1 h. The optical density was then adjusted to OD₆₀₀ of 0.7 prior to infiltration. The suspension was infiltrated into the abaxial side of leaves using a needleless syringe. Leaves infiltrated with empty vector with no actRNA served as negative controls.

### Luciferase assay

Leaf discs from infiltrated leaves were collected at three- and six-days post-infiltration. Proteins were extracted using a modified protocol from Herrera-Estrella et al. [24]. Briefly, the leaves were manually ground with tissue grinders in a modified Luc buffer (50 mM Na-phosphate pH 7.0; 4% soluble PVP [MW 360,000]; 2 mM EDTA; 20 mM DTT). The supernatant was then used to perform the dual luciferase assay according to the manufacturer’s instructions using the Dual-Glo^®^ Luciferase Assay System (Promega).

### Statistical analysis

Statistical analyses were performed using Origin (OriginPro 2024). Pairwise two-tailed Welch’s t-tests comparing each actRNA condition to the no-actRNA control. The number of biological replicates (n) is indicated for each experiment; either four or six replicates were measured. Statistical significance was evaluated at three thresholds; *p* < 0.05, *p* < 0.01, and *p* < 0.005. Data are presented as mean ± standard error (SE).

## Results

### Transcription regulation by the CRISPRa/d*Sa*Cas9 system

To establish a modular CRISPRa system compatible with multiple Cas9 orthologs, we cloned both CRISPRa/d*Sa*Cas9 and CRISPRa/d*Sp*Cas9 constructs using the hierarchical multi-kingdom Golden Gate cloning platform. As a test system, we selected the *pNOS* promoter to drive *firefly luciferase* (*FLuc*) expression, targeted by dCas9:EDLL effectors. Six guide RNAs (gRNAs) were designed to bind within the *pNOS* promoter region, each flanked by the *Sa*Cas9-specific PAM sequence (5′-NNGRRT-3′). These gRNAs were assembled with the *Sa*Cas9 trans-activating CRISPR RNA (tracrRNA) and an MS2 scaffold to generate activation RNAs (actRNAs), all expressed under the *Arabidopsis thaliana* U6-26 promoter.

**Fig. 2:**
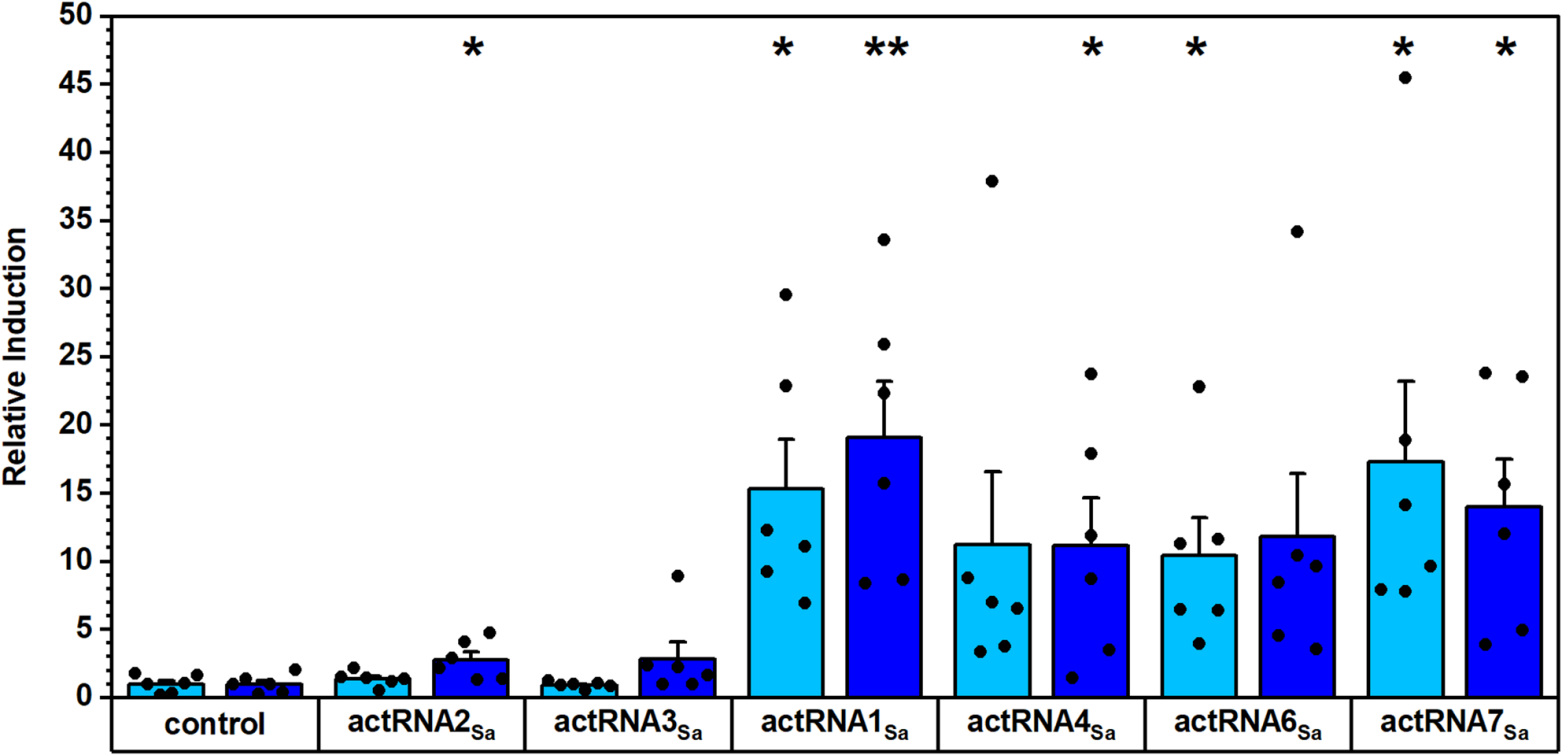
Transcriptional activation of the *pNOS* promoter by d*Sa*Cas9 in *Nicotiana benthamiana* using different actRNAs. Transient expression assays were performed in *N. benthamiana* leaves co-infiltrated with Agrobacterium strains carrying constructs for d*Sa*Cas9, MCP:VPR, and individual actRNAs targeting the *pNOS* promoter driving firefly luciferase (FLuc). *Renilla* luciferase (RLuc), driven by a *p35S* promoter, served as an internal control. Luciferase activity was measured at 3 days post-infiltration (dpi; light blue bars) and 6 dpi (dark blue bars) using a dual-luciferase assay. Data represent FLuc/RLuc ratios normalized to the no-actRNA control. Individual data points (dots), mean values (bars), and standard errors (error bars) are shown (*n* = 6; pairwise two-tailed Welch’s t-tests comparing each actRNA condition to the no-actRNA control; *p < 0.05; **p < 0.01; ***p < 0.005)

The complete constructs were transformed into *A. tumefaciens* strain EH105 and infiltrated into *N. benthamiana* leaves. Leaf samples were collected at 3- and 6-days post-infiltration (dpi) for dual-luciferase assays. Firefly luciferase (FLuc) luminescence was normalized to *Renilla* luciferase (RLuc), expressed from a *p35S* promoter, and subsequently scaled to the mean signal intensity of the control samples lacking act-RNA (Fig. 2).

Among the six actRNAs tested, actRNA2_Sa_ and actRNA3_Sa_, both targeting the 3′ region of the promoter, did not induce detectable promoter activity at either time point (Fig. 2). In contrast, actRNA1_Sa_, actRNA4_Sa_, actRNA6_Sa_, and actRNA7_Sa_ activated the *pNOS* promoter, with 15.3-, 11.2-, 10.4-, and 17.3-fold induction at 3 dpi, respectively (Fig. 2, light grey bars). At 6 dpi, actRNA1_Sa_ showed further increased induction (19-fold), while actRNA4_Sa_ and actRNA6_Sa_ maintained similar levels. A slight decrease was observed for actRNA7_Sa_ (14-fold), though this change was not statistically significant.

**Fig. 3:**
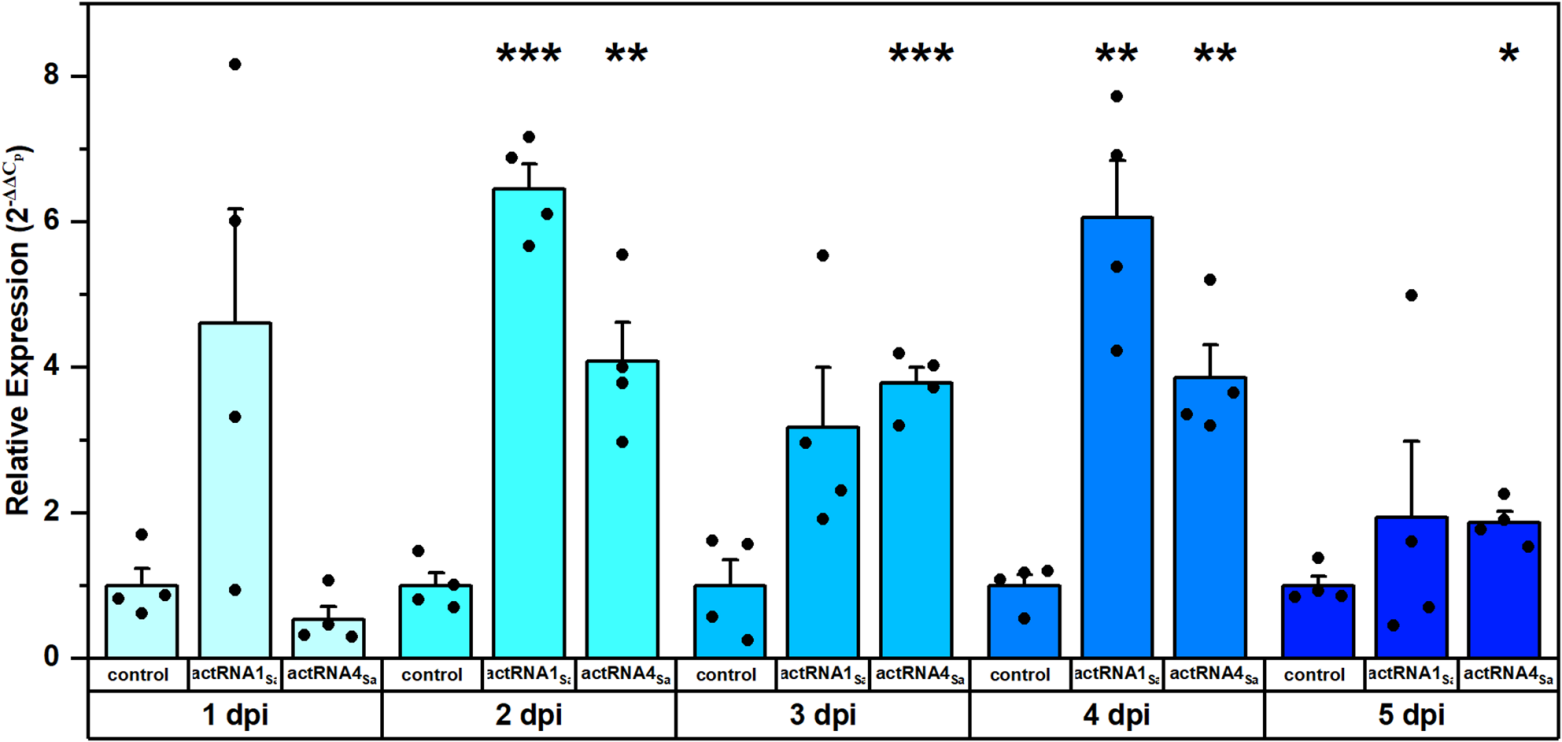
Time-course analysis of luciferase expression in *Nicotiana benthamiana* leaves using RT-qPCR. Samples were collected at multiple time points post-infiltration from *N. benthamiana* leaves transiently expressing d*Sa*Cas9, MCP:VPR, and individual actRNAs targeting the *pNOS* promoter. Total RNA was extracted and reverse-transcribed to cDNA for quantitative analysis. Firefly luciferase (FLuc) and *Renilla* luciferase (RLuc) transcript levels were quantified using gene-specific primers. FLuc expression was double-normalized: first to RLuc expression, and then to the FLuc/RLuc ratio of the no-actRNA control. Data points represent individual biological replicates (dots), with mean values (bars) and standard errors (error bars) shown (*n* = 4; pairwise two-tailed Welch’s t-tests comparing each actRNA condition to the no-actRNA control; *p < 0.05; **p < 0.01; ***p < 0.005)

To assess transcriptional activation at the mRNA level, we performed RT-qPCR on samples collected from 1 to 5 dpi using actRNA1_Sa_, actRNA4_Sa_, and a no-actRNA control. FLuc transcript levels were normalized to RLuc. The actRNA1_Sa_ induced elevated FLuc expression at 1, 2, and 4 dpi, with a transient dip at 3 dpi and a return to near-control levels by 5 dpi (Fig. 3). The actRNA4_Sa_ did not show increased expression at 1 dpi but maintained stable induction from 2 to 4 dpi, followed by a decline at 5 dpi. These results confirm that the CRISPRa/d*Sa*Cas9 system can robustly activate gene expression in a time- and target site-dependent manner.

### Direct comparison of the CRISPRa/d*Sa*Cas9 and the CRISPRa/d*Sp*Cas9 using a shared target site

In this study the developed modular CRISPRa/Cas ortholog system enables the use of both d*Sa*Cas9 and d*Sp*Cas9 effectors, providing a unique opportunity for direct functional comparison. However, such comparisons are constrained by the availability of shared target sites within the promoter region, as proximity and sequence compatibility are critical for meaningful evaluation. Analysis of the *pNOS* promoter revealed a conserved target site that is suitable for recognition by orthologous factors across species. The actRNA1_*Sa*_, originally designed for d*Sa*Cas9, was found to also function with d*Sp*Cas9 and was designated actRNA12_*Sp*_. Notably, the 3′ end of the target sequence contains the motif 5′-TGGAGT-3′, which satisfies the PAM requirements for both *Sa*Cas9 (5′-NNGRRT-3′) and *Sp*Cas9 (5′-NGG-3′).

**Fig. 4:**
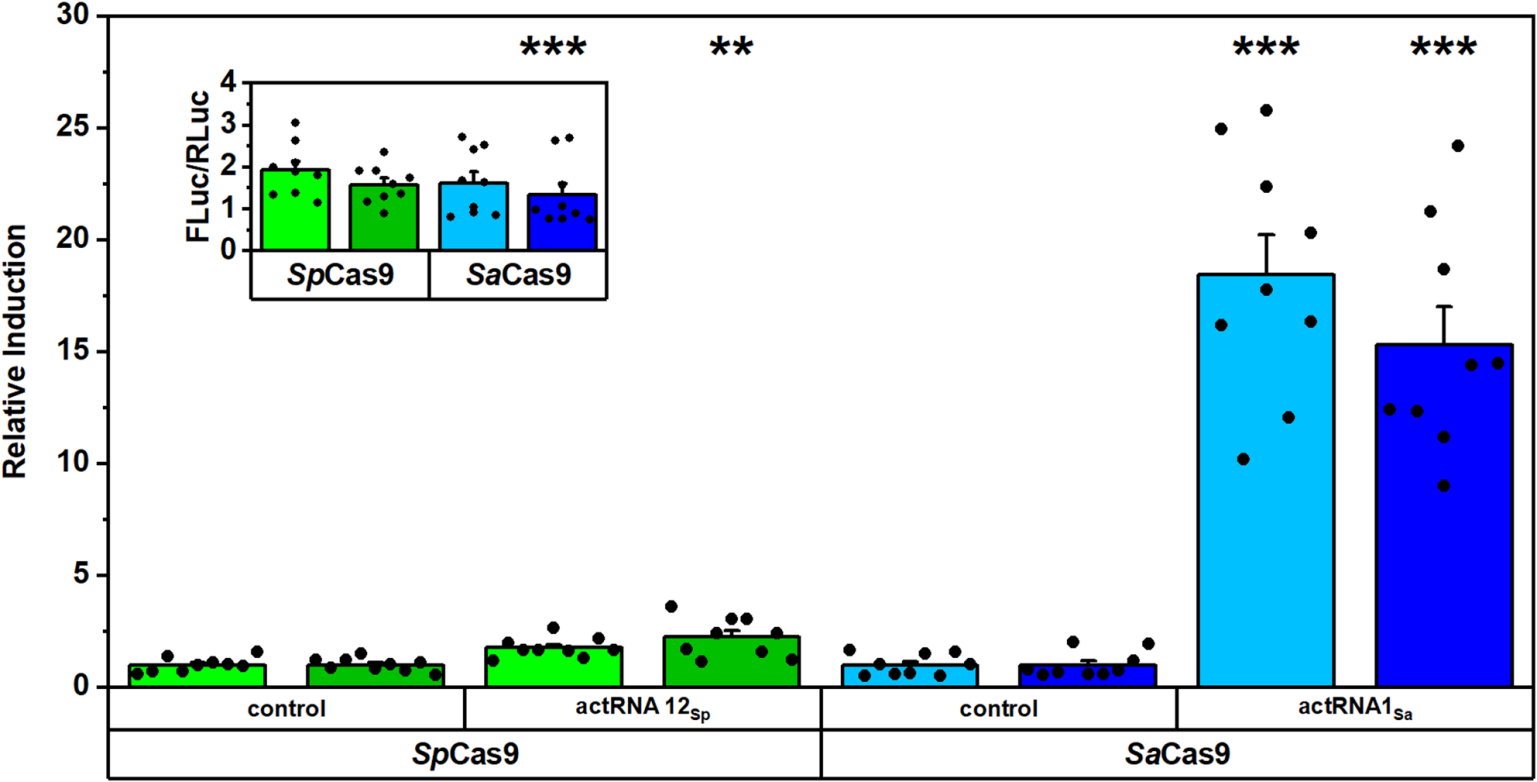
Comparative analysis of promoter activation by d*Sp*Cas9 and d*Sa*Cas9 in *Nicotiana benthamiana*. The two dCas9 orthologs, d*Sp*Cas9 and d*Sa*Cas9, were tested for their ability to activate the *pNOS* promoter using a shared target site. Constructs expressing either d*Sp*Cas9 or d*Sa*Cas9 were infiltrated into *N. benthamiana* leaves with or without their respective actRNAs (actRNA12_Sp_ or actRNA1_Sa_ and control, respectively). Firefly luciferase (FLuc) expression, driven by the *pNOS* promoter, was normalized to *Renilla* luciferase (RLuc) expressed from a *p35S* promoter. Luciferase activity was measured at 3 days post-infiltration (dpi; light green and blue bars for d*Sp*Cas9 and d*Sa*Cas9, respectively) and 6 dpi (dark green and blue bars for d*Sp*Cas9 and d*Sa*Cas9, respectively) using a dual-luciferase assay. Data represent relative induction normalized to the corresponding construct lacking actRNA. Insert: absolute FLuc/RLuc ratios for constructs without actRNA. Individual data points (dots), mean values (bars), and standard errors (error bars) are shown (*n* = 6; pairwise two-tailed Welch’s t-tests comparing each actRNA condition to the no-actRNA control; *p < 0.05; **p < 0.01; ***p < 0.005)

The basal expression levels, measured as FLuc/RLuc ratios in the absence of actRNA, were comparable between the d*Sa*Cas9 and d*Sp*Cas9 systems, ranging from 1.35 ± 0.26 to 1.93 ± 0.21 (Fig. 4, insert). This consistency indicates that the background activity of the CRISPRa system is independent of the dCas9 ortholog used, and that neither dCas9 variant alone significantly influences reporter gene expression without actRNA-mediated targeting. The CRISPRa/d*Sa*Cas9 system induced statistically significant promoter activation when the actRNA targeting the shared site within the *pNOS* promoter was co-expressed. In contrast, the CRISPRa/d*Sp*Cas9 system yielded modest but statistically significant induction levels of 1.76 ± 0.15 and 2.24 ± 0.29 at 3 and 6 dpi, respectively (Fig. 4). CRISPRa/d*Sa*Cas9, however, induced substantially higher activation levels of 18.44 ± 1.80 and 15.32 ± 1.67 at the same time points. These results demonstrate that, although both orthologues are capable of activating transcription from the same target site, d*Sa*Cas9 exhibits markedly higher activation efficiency, highlighting its potential as a more effective CRISPRa effector in transient plant expression systems.

### CRISPRa/d*Sp*Cas9 dependent promoter induction

**Fig. 5:**
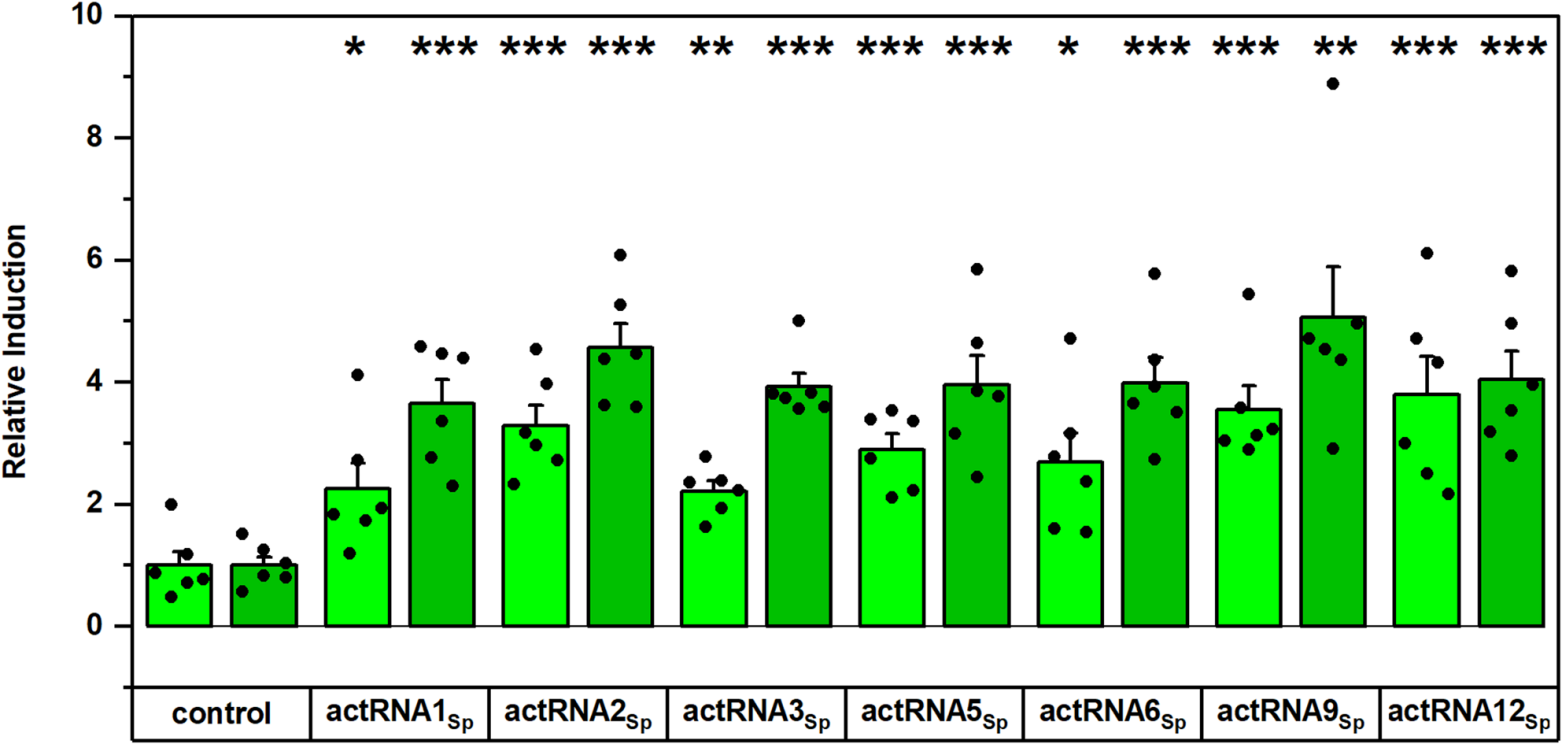
Transcriptional activation of the *pNOS* promoter by d*Sp*Cas9 in *Nicotiana benthamiana* using different actRNAs. Transient expression assays were performed in *N. benthamiana* leaves infiltrated with *Agrobacterium tumefaciens* strains carrying constructs for d*Sp*Cas9, MCP:VPR, and individual actRNAs targeting the *pNOS* promoter upstream of a firefly luciferase (FLuc) reporter. *Renilla* luciferase (RLuc), driven by a *p35S* promoter, served as an internal control. Luciferase activity was measured at 3 days post-infiltration (dpi; light green bars) and 6 dpi (dark green bars) using a dual-luciferase assay. Data represent FLuc/RLuc ratios normalized to the no-actRNA control. Individual data points (dots), mean values (bars), and standard errors (error bars) are shown (*n* = 6; pairwise two-tailed Welch’s t-tests comparing each actRNA condition to the no-actRNA control; *p < 0.05; **p < 0.01; ***p < 0.005)

To further evaluate the performance of the modular CRISPRa/Cas ortholog system, we tested additional actRNAs targeting the *pNOS* promoter using d*Sp*Cas9. After cloning the corresponding actRNAs into final expression constructs, each was individually infiltrated into *N. benthamiana* leaves. Luciferase activity was measured at 3 and 6 dpi using a dual-luciferase assay. Consistent with previous results for actRNA12_*Sp*_, the overall induction of the *pNOS* promoter by d*Sp*Cas9 (Fig. 5) was lower than that observed with d_Sa_Cas9.

At 6 dpi, all tested actRNAs targeting the *pNOS* promoter with d*Sp*Cas9 showed statistically significant induction compared to the no-actRNA control. However, at 3 dpi, actRNA1_*Sp*_, actRNA3_*Sp*_, and actRNA6_*Sp*_ did not reach significance. These findings support the notion that *Sa*Cas9, previously reported to exhibit higher mutation frequencies in genome editing applications, may also outperform *Sp*Cas9 in transcriptional activation in plant systems. This superior mutation frequency is explained by the smaller size of *Sa*Cas9 compared to *Sp*Cas9 which facilitates a more efficient nuclear import [25, 26] as well as an enhanced DNA binding dynamic [27]. It should be noted that the induction levels observed in our system were lower than those reported in other CRISPRa/d*Sp*Cas9 studies. For example, actRNA12_*Sp*_ which is targeting a site previously validated [4] induced only a 4-fold increase in our system, whereas up to 12-fold induction has been reported by Selma et al. [4]. To investigate this discrepancy, we compared the construct architectures and identified the *pNOS* promoter used in our system was substantially longer than the truncated promoter used in published systems as a key difference.

To assess whether promoter length influenced activation efficiency, we cloned the pNOS_trc_ into our CRISPRa system. Constructs containing *pNOS*_*trc*_ and actRNAs (actRNA1_*Sp*_, actRNA3_*Sp*_, actRNA5_*Sp*_, and actRNA12_*Sp*_) were tested alongside a no-actRNA control. At both 3 and 6 dpi, d*Sp*Cas9 in combination with these actRNAs induced stronger activation of the *pNOS*_*trc*_ promoter compared to the full-length version (Suppl. Figure 1). Notably, actRNA12_*Sp*_ achieved approximately 6-fold induction with *pNOS*_*trc*_. This remains a lower induction compared to the 12-fold induction shown previously by Selma et al. [4]. It should be noted that the absolute FLuc/RLuc luminescence values for *pNOS*_*trc*_ were markedly lower than those for the full-length promoter (Suppl. Figure 2, insert), indicating reduced basal expression. These results suggest that promoter architecture significantly influences CRISPRa efficiency and that truncated promoters may enhance relative induction while reducing absolute expression levels. This highlights the importance of promoter selection in CRISPRa system design and benchmarking.

### Effect of multiple actRNAs on transcriptional activation

In addition to promoter architecture, previous studies have explored whether expressing multiple copies of the same actRNA or combining different actRNAs can enhance CRISPRa-mediated gene activation (Pan et al., 2021). To investigate this in our system, we constructed expression cassettes containing either two or three multiplexed copies of actRNA12_*Sp*_ or actRNA1_*Sa*_. These constructs were assembled with their respective Cas9 orthologs and introduced into *N. benthamiana* leaves. Luciferase activity was measured 6 dpi using a dual-luciferase assay.

**Fig. 6:**
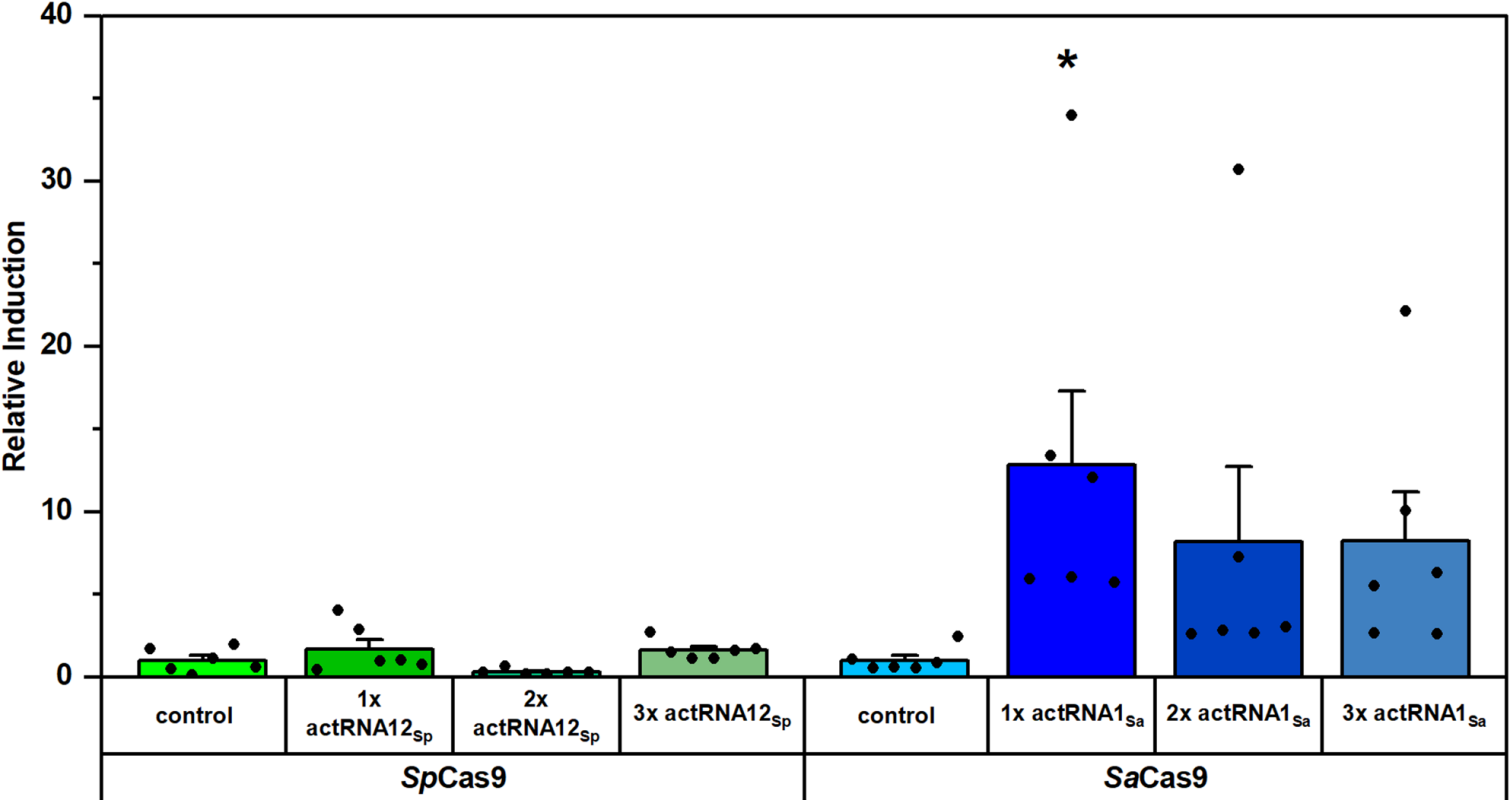
Promoter activation by multiplexed expression of actRNAs in *Nicotiana benthamiana*. Multiplexed expression cassettes containing multiple copies of actRNAs targeting the *pNOS* promoter were constructed for both d*Sp*Cas9 and d*Sa*Cas9 systems using the shared target site actRNAs. *N. benthamiana* leaves were infiltrated with the respective constructs, and luciferase activity was measured at 6 days post-infiltration (dpi) using a dual-luciferase assay. Firefly luciferase (FLuc) expression was normalized to *Renilla* luciferase (RLuc) and further normalized to the no-actRNA control. Data points represent individual biological replicates (black dots), with mean values (bars) and standard errors (error bars) shown (*n* = 6; pairwise two-tailed Welch’s t-tests comparing each actRNA condition to the no-actRNA control; *p < 0.05; **p < 0.01; ***p < 0.005)

The construct containing two copies of actRNA12_*Sp*_ resulted in reduced promoter activation compared to the no-actRNA control. The construct containing three copies restored induction to a level comparable to the single-copy construct (Fig. 6). For d*Sa*Cas9, both the two-copy and three-copy actRNA1_*Sa*_ constructs exhibited lower induction than the single-copy version. These results suggest that increasing the number of identical actRNA copies does not enhance transcriptional activation in the current experimental setup (Fig. 6).

To assess whether combining distinct actRNAs could improve activation, we cloned actRNA3_*Sp*_, actRNA5_*Sp*_, and actRNA12_*Sp*_ into separate expression cassettes and assembled all possible combinations into final constructs. These were tested in *N. benthamiana* leaves at 3 and 6 dpi. None of the combinations produced significantly higher induction than the individual actRNA constructs (Supplementary Fig. 3).

These findings suggest that, within the context of our modular CRISPRa/Cas ortholog system, expressing multiple copies of the same actRNA or combining different actRNAs does not enhance promoter induction. Even though it has not been shown for the *pNOS* or *pNOS*_*trc*_ promoter, previous studies have confirmed that targeting a promoter with multiple actRNAs can enhance the expression of target genes [4, 28].

## Discussion

Here we present for the first time a modular CRISPRa/Cas ortholog system designed for flexible and comparative transcriptional activation studies in plants. Built on the multi-kingdom Golden Gate cloning platform [10, 11], this system enables rapid assembly of CRISPRa components and supports the use of multiple Cas9 orthologs. Using transient expression in *N. benthamiana*, we systematically evaluated the performance of d*Sa*Cas9 and d*Sp*Cas9 in activating the *pNOS* promoter. To our knowledge the most used Cas9 ortholog is the CRISPRa/d*Sp*Cas9, while CRISPRa/d*Sa*Cas9 is not described in any plant species yet, despite its well-known higher mutation frequencies for CRISPR-based mutagenesis [1, 2].

Our results demonstrate that d*Sa*Cas9 consistently surpasses d*Sp*Cas9 in transcriptional activation in transient plant dual-luciferase assay. Among six actRNAs tested with d*Sa*Cas9, four induced strong promoter activation, achieving up to a 19-fold increase in expression. In contrast, d*Sp*Cas9 resulted in only modest activation up to 5-fold, even when targeting the same site as d*Sa*Cas9. These findings align with previous reports of higher editing efficiency for *Sa*Cas9 in plant systems [1, 2]. The enhanced efficiency observed with *Sa*Cas9 is likely attributable to its smaller size, improved nuclear localization, and more restrictive PAM requirements, which may also reduce off-target effects. Moreover, in mice a CRISPRa/d*Sa*Cas9 has been used and, interestingly, the CRISPRa/d*Sa*Cas9 system demonstrated superior performance compared to the CRISPRa/d*Sp*Cas9 system, inducing approximately a twofold stronger activation in mice [20].

Although we observed stronger transcriptional activation of the pNOS promoter, CRISPRa systems based on this orthologue could be constrained by PAM availability at endogenous plant promoters. To assess whether PAM stringency poses a practical limitation for future applications, we bioinformatically analysed PAM distribution across endogenous tomato promoters of genes encoding phenylalanine ammonia-lyase (PAL), caffeoyl-CoA *O*-methyltransferase (CCoAOMT), and 4-coumarate CoA ligase (4CL) from phenylalanine pathway (Supplementary Table S3). This analysis compared *Sp*Cas9 and *Sa*Cas9, the natural *Streptococcus thermophilus* Cas9 ortholog *Sth1*Cas9, as well as PAM-expanded and -relaxed variants of both *Sp*Cas9 and *Sa*Cas9. While the stricter NNGRRT PAM of *Sa*Cas9 and the NNAGAAW PAM of *Sth1*Cas9 reduce the absolute number of targetable sites relative to SpCas9, sufficient PAM-compatible positions were consistently present across all promoters analysed (Supplementary Table S3). Moreover, PAM-relaxed variants such as *Sa*Cas9-KKH and *Sp*Cas9-derived SpG substantially increased target site density without promoter modification, and PAM-expanded variants like *Sp*Cas9-VQR and -EQR further extended promoter accessibility. These observations indicate that the CRISPRa strategy demonstrated here using *pNOS* is transferable to endogenous plant promoters and that PAM availability can be actively addressed at the design stage through Cas9 orthologue selection or the use of PAM-relaxed variants, rather than constituting a prohibitive constraint.

More broadly, the selection of a dCas9 orthologue involves balancing transcriptional potency with practical targetability in plant genomes, where PAM availability and guide architecture impose non-trivial constraints [29, 30]. In this study, d*Sa*Cas9:EDLL consistently outperformed d*Sp*Cas9:EDLL under identical recruitment logic, and direct comparison at a shared promoter target site confirms that this difference is not attributable solely to guide positioning. In this context, PAM recognition should be considered a design parameter rather than a fixed limitation. PAM-relaxed variants such as *Sa*Cas9-KKH or *Sp*Cas9-derived SpG provide a practical compromise between targeting flexibility and specificity, whereas near-PAM-less enzymes such as SpRY, despite expanding potential target space, complicate promoter-focused guide design and specificity control [31, 32]. In parallel, protein engineering and machine-learning approaches increasingly enable the generation of Cas9 variants with tailored PAM requirements, allowing selective expansion of targetable sites without globally relaxed PAM recognition [33–35]. Within the scope of this work, these considerations support prioritising dCas9 orthologue performance while introducing PAM adaptations only when required for specific endogenous targets.

Beyond PAM considerations, MCP/MS2-based CRISPRa systems introduce an additional layer of control through RNA scaffold engineering. RNA aptamer-mediated effector recruitment constitutes a modular design axis that is largely decoupled from Cas protein optimisation [30, 36, 37]. Multiple orthogonal RNA-protein modules, such as MS2/MCP and PP7/PCP, allow parallel recruitment of distinct effectors, and multi-aptamer scaffolds have been shown to modulate effector stoichiometry and transcriptional output [36]. In the present study, RNA scaffold architecture was deliberately held constant to enable direct assessment of orthologue-dependent CRISPRa performance, while preserving compatibility with future RNA scaffold optimisation.

In addition to RNA-scaffold-based SAM-like systems, alternative CRISPRa architectures have been developed for plants, including CRISPR-Act3.0 and epitope-based recruitment strategies such as SunTag and MoonTag [30, 38]. These approaches share the goal of enhancing transcription through multivalent activator recruitment but differ in implementation: SAM-derived and CRISPR-Act3.0 systems recruit auxiliary activators via RNA aptamer-coat protein interactions, whereas SunTag- and MoonTag-based systems amplify signal through peptide repeat arrays fused directly to dCas9 [4, 30, 38]. Architecture-level benchmarking in plants has so far focused on such recruitment strategies, primarily using d*Sp*Cas9 [4]. In contrast, the present study compares Cas9 orthologues under matched CRISPRa conditions, using a single-plasmid SAM-like architecture, a full-length *pNOS* promoter, and defined component stoichiometry including p19. This design enables direct evaluation of orthologue-dependent performance without confounding effects from delivery or recruitment architecture.

From a mechanistic perspective, the lack of enhanced activation upon increasing actRNA copy number suggests that CRISPRa output in this configuration is not limited by guide abundance once stable dCas9-gRNA-DNA complexes are formed [39]. Owing to the large DNA footprint and slow unbinding kinetics of dCas9, promoter engagement can approach saturation, such that additional guides do not yield proportional increases in regulatory output [40]. Under these conditions, increasing actRNA number may instead increase competition for a shared dCas9 pool, leading to dilution of effective activator binding. In our multiplexing experiments, combining multiple actRNAs targeting *pNOS* did not produce additive activation (Fig. 6; Supplementary Fig. 3), consistent with both resource-limited behaviour and steric interference between neighbouring dCas9 complexes. Experimental work has shown that such interference can occur at guide spacings of approximately 10–20 bp, depending on orientation and promoter context [40]. Together, these observations underscore that multiplexed CRISPRa performance depends strongly on guide spacing and promoter architecture, and that increasing guide number alone is not predictive of improved transcriptional output [41].

Finally, comparison of luciferase transcript and activity levels confirmed transcriptional activation by CRISPRa/dSaCas9 but revealed stronger induction at the reporter activity level than at the mRNA level. Such discrepancies are well documented and reflect differences in translation efficiency, protein stability, and signal accumulation intrinsic to enzymatic reporter assays [42, 43]. Because all constructs were evaluated using the same reporter architecture and assay conditions, this divergence does not affect the comparative interpretation of orthologue-dependent CRISPRa performance.

Direct comparison of d*Sa*Cas9 and d*Sp*Cas9 at a shared promoter target site further confirmed the higher activation potential of d*Sa*Cas9. While both orthologs were capable of inducing expression, d*Sp*Cas9 achieved only a two-fold increase, compared to a significantly stronger response from d*Sa*Cas9 of 19-fold. This highlights the value of our system in enabling side-by-side benchmarking of Cas9 variants under identical experimental conditions. Our finding aligns with similar results in mice where it has been shown that the CIRSPRa/d*Sa*Cas9 results in a higher gene expression by the target promoter [20]. In other studies, using the d*Sp*Cas9, it has been shown that the transcriptional expression of different promoters induced by the system itself is ranging between 4- to 12-fold [4, 28].

Interestingly, the activation efficiency of d*Sp*Cas9 in our system was lower than that reported in other studies using similar constructs, especially the one of Selma et al. [4, 9]. In their work Selma et al. [4] developed the CRISPRa/d*Sp*Cas9 system based on a d*Sp*Cas9:EDLL effector and an MCP:VPR effector. We have adapted this dual effector system, including the spontaneous mutation in the MS2 scaffold that occurred during their cloning process [4]. Upon closer examination, we identified promoter architecture as a key variable. The *pNOS* promoter used in our constructs was substantially longer than the truncated promoter used in their published system. When we tested a truncated version (*pNOS*_*trc*_), we observed a lower level of basal expression of the promoter, leading to an increased relative induction by the CRISPRa/Cas9 system.

A further important factor explaining the lower activation efficiency observed in our system compared to Selma et al. [4] is the difference in plasmid delivery strategy. While our approach utilizes a single plasmid containing both the CRISPRa machinery and the dual-luciferase reporters, Selma et al. [4] employed co-infiltration of two separate plasmids, one for the dual-luciferase reporter and one for the CRISPRa system [6, 44]. Recent systematic analyses in *N. benthamiana* have demonstrated that the method of construct delivery can significantly influence both the mean expression levels and the variability of reporter gene assays [45]. For example, Tang et al. [45] showed that co-infiltration of separate plasmids can result in a subset of cells receiving higher doses of the activator, leading to higher maximal induction but also increased variability, whereas single-plasmid delivery ensures stoichiometric delivery of all components, potentially reducing variability but also limiting peak induction due to plasmid size or transcriptional competition. However, it should be noted that larger plasmids often exhibit reduced expression efficiency due to increased structural complexity, including higher DNA topology, which can reduce transcriptional accessibility. Additionally, larger plasmids may experience impaired nuclear import, which limits their effective delivery and expression in plant cells. Despite these challenges, the Multi-Kingdom Golden-Gate system enables the assembly such large single plasmids with all required CRISPRa components [10, 11], including the dCas9 effector, the MCP:VPR effector, the actRNA scaffolds, and the reporter genes, offering a streamlined and modular approach for transcriptional activation studies transient in plants and in future also for stable transformation into plant cells.

## Conclusion

In conclusion, this system provides a robust platform for testing and comparing CRISPRa components in a standardized manner. The ability to achieve up to four-fold higher induction with CRISPRa/d*Sa*Cas9 compared to CRISPRa/d*Sp*Cas9 enables more sensitive detection and fine-tuning of promoter activation. While high induction is desirable for many applications, the option to use either d*Sa*Cas9 or d*Sp*Cas9 also allows for tailored modulation of gene expression, depending on experimental needs. Future work may extend this system to additional Cas orthologs, such as the more compact *Campylobacter jejuni* Cas9 or the highly active *Streptococcus thermophilus* Cas9 in plants or explore its application in stable transformation systems and tissue-specific expression contexts. By providing a flexible and modular system, we aim to accelerate the development and deployment of CRISPRa technologies in plant biotechnology.

## Supplementary Information

Supplementary Material 1.

## Data Availability

All data generated or analyzed during this study are included in this published article and its supplementary information files.
