# Supplementary material for: dSaCas9 enables enhanced transcriptional activation in Nicotiana benthamiana compared to its dSpCas9 ortholog
Source: BMC Plant Biol. 2026 Jun 4;26:1338. doi: 10.1186/s12870-026-09174-6 (PMC13455137; doi:10.1186/s12870-026-09174-6)
Supplement: Supplementary file 1 [file 12870_2026_9174_MOESM1_ESM.pdf]

Supplementary Information

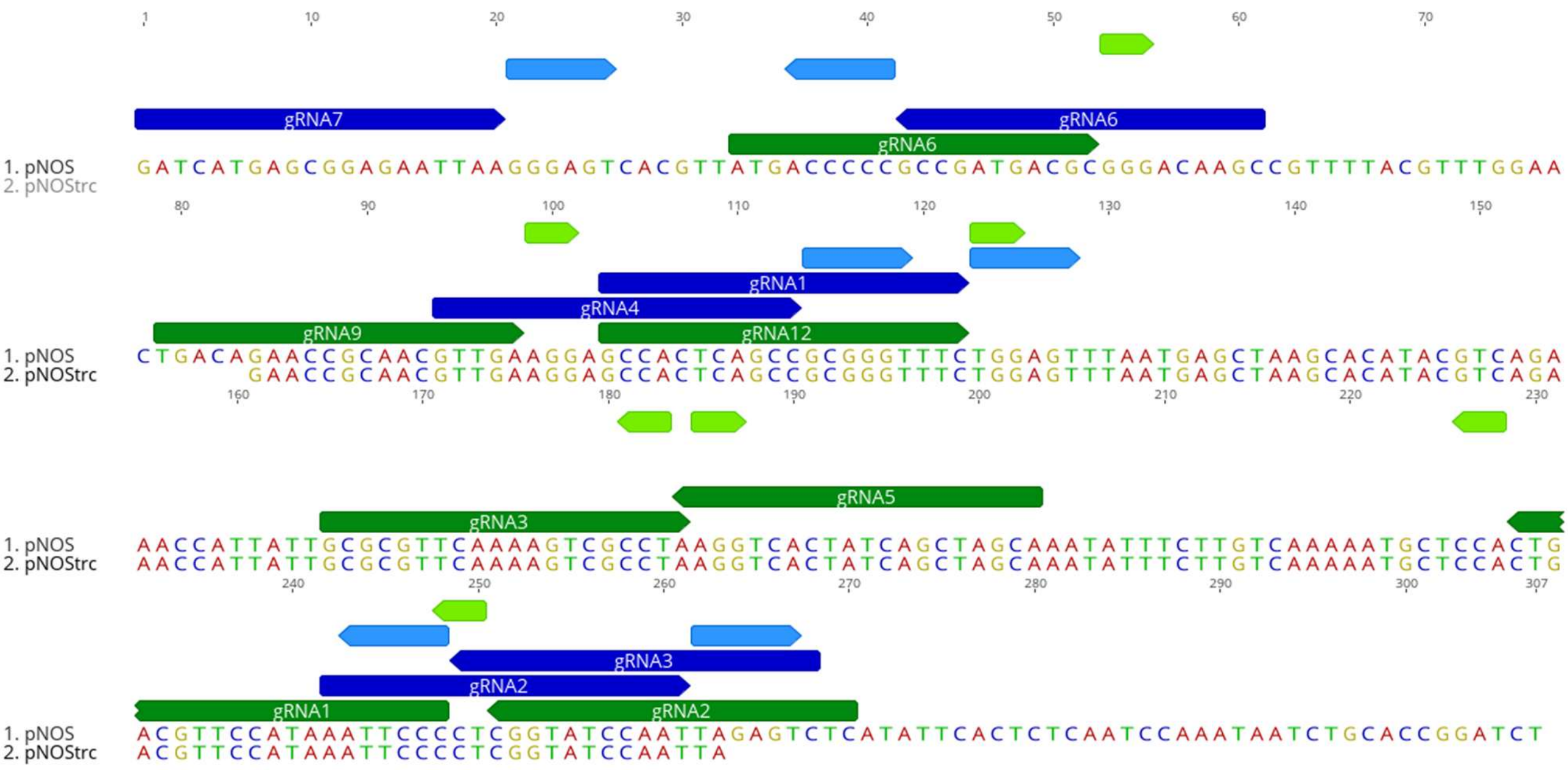

Suppl. Figure 1: Architecture of target promoters and gRNAs. Shown is the sequence alignment of the *pNOS* promoter and the *pNOS*$_{trc}$ promoter. In addition, the gRNAs leading to the used actRNAs for d*Sa*Cas9 are shown in dark blue and for d*Sp*Cas9 in dark green. While the corresponding PAM sites are highlighter in light blue and light green for d*Sa*Cas9 and d*Sp*Cas9, respectively.

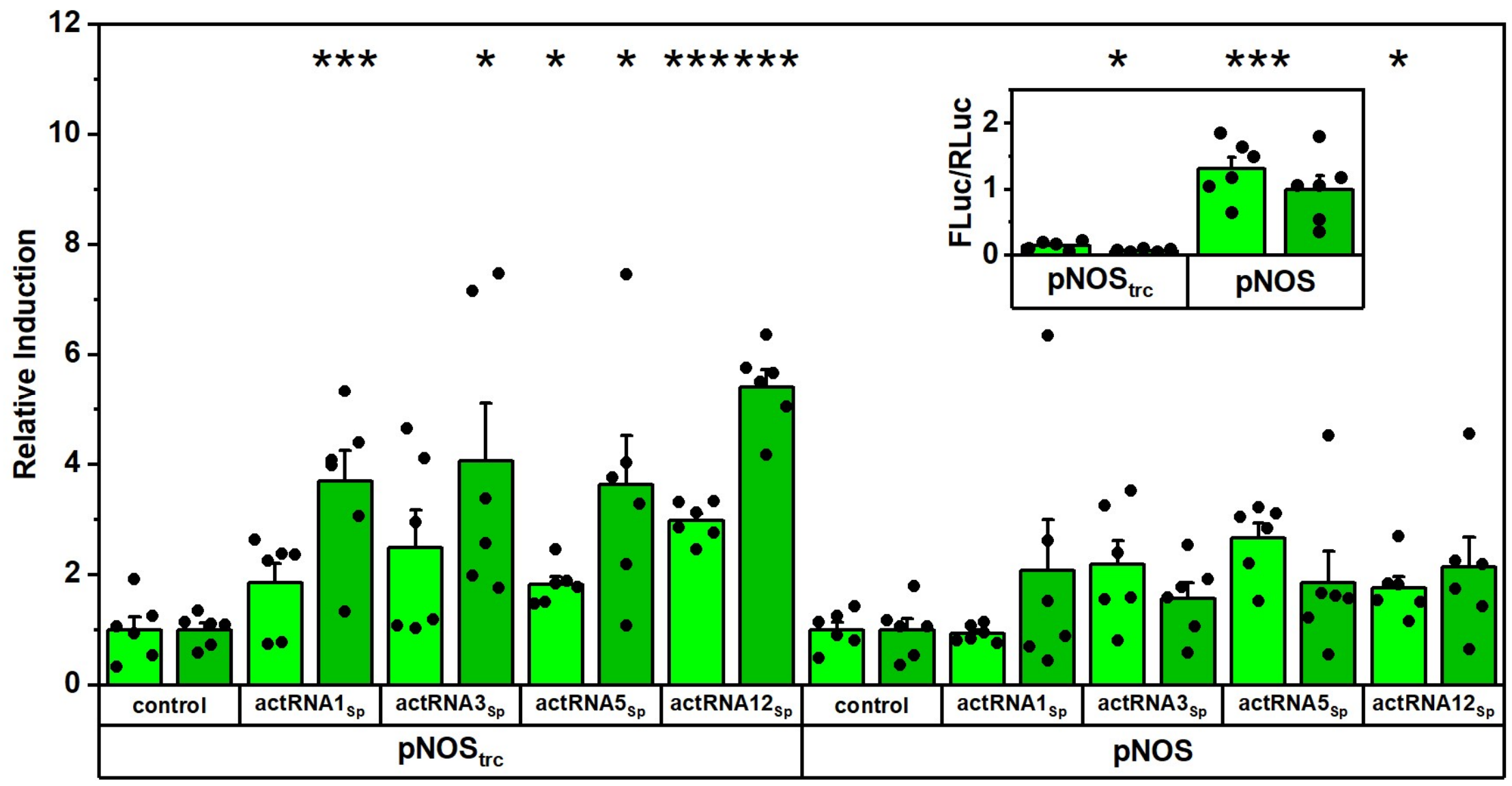

Suppl. Figure 2: Comparative activation of full-length and truncated *pNOS* promoters by d*Sp*Cas9 in *Nicotiana benthamiana*. Transient expression assays were conducted in *N. benthamiana* leaves infiltrated with constructs expressing d*Sp*Cas9, MCP:VPR, and different actRNAs targeting either a truncated truncated version (*pNOStrc*) or the full-length *pNOS* promoter. Firefly luciferase (FLuc) expression, driven by each promoter, was normalized to *Renilla*

luciferase (RLuc) expressed from a *p35S* promoter. Luciferase activity was measured at 3 days post-infiltration (dpi; light green bars) and 6 dpi (dark green bars) using a dual-luciferase assay. Data represent FLuc/RLuc ratios normalized to the no-actRNA control. Individual data points (dots), mean values (bars), and standard errors (error bars) are shown. Inset: absolute FLuc/RLuc values for constructs lacking actRNAs. (Inset) Shown is the absolute FLuc/RLuc for the non-actRNA controls (n = 6; pairwise two-tailed Welch's t-tests comparing each actRNA condition to the no-actRNA control; *p < 0.05; **p < 0.01; ***p < 0.005)

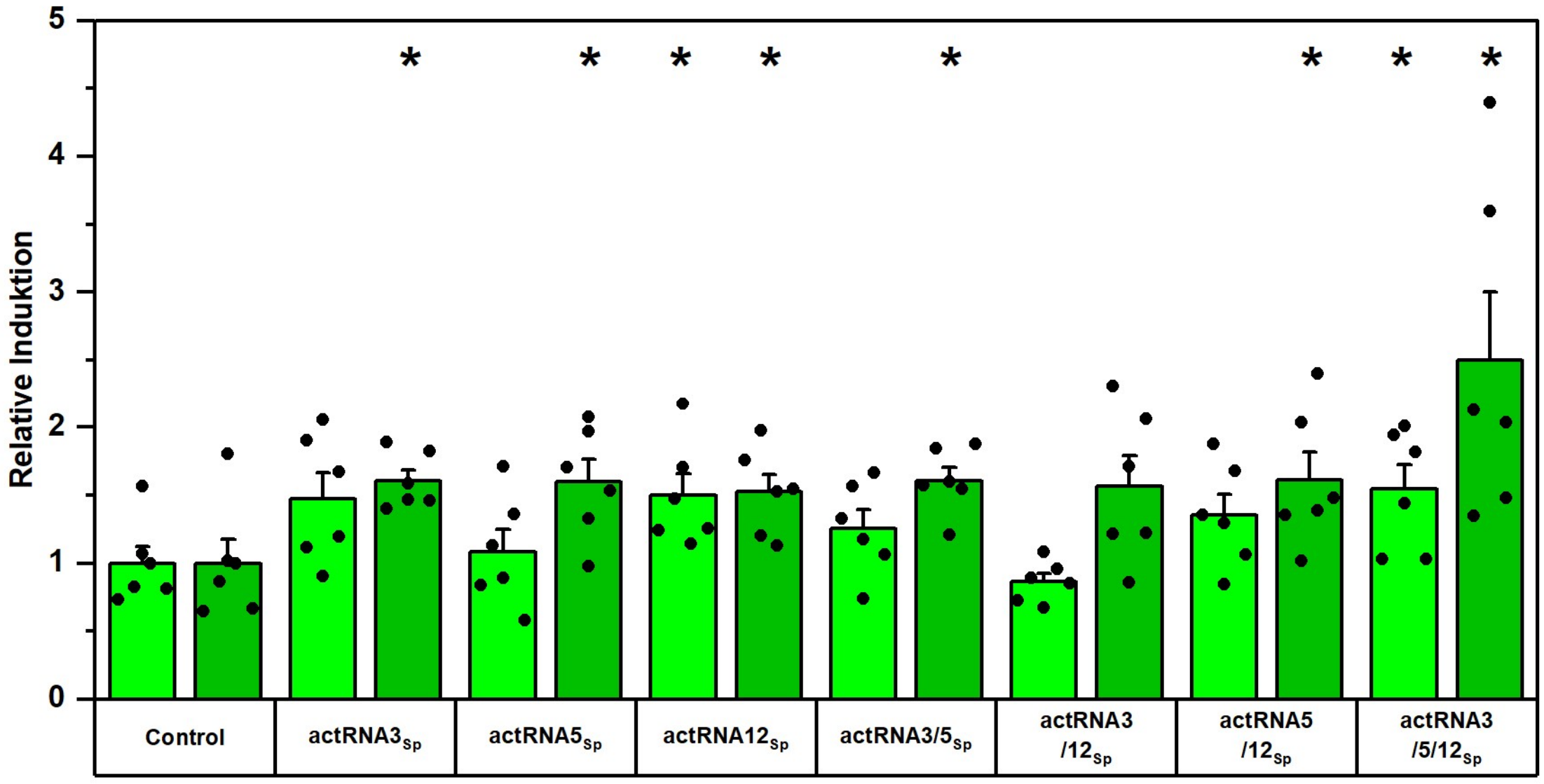

Suppl. Figure 3: Effect of actRNA co-expression on *pNOS* promoter activation in *Nicotiana benthamiana*. To evaluate the impact of co-expression of actRNA, *N. benthamiana* leaves were infiltrated with constructs expressing d*Sp*Cas9, MCP:VPR, and either individual or combinatorial actRNAs targeting the *pNOS* promoter. Luciferase activity was measured at 3 dpi (light green bars) and 6 dpi (dark green bars) using a dual-luciferase assay. Firefly luciferase (FLuc) expression was normalized to *Renilla* luciferase (RLuc) and further normalized to the no-actRNA control. Data represent FLuc/RLuc ratios for each actRNA configuration. Individual data points (black dots), mean values (bars), and standard errors (error bars) are shown (n = 6; pairwise two-tailed Welch's t-tests comparing each actRNA condition to the no-actRNA control; *p < 0.05; **p < 0.01; ***p < 0.005).

Supplementary Table S1. DNA and RNA sequences used in this study. Sequences of all DNA and RNA elements used for construction of the CRISPRa system and reporter assays are listed. Individual functional elements within each sequence are colour-coded for clarity. Green indicates promoter regions, blue denotes coding sequences, purple marks activation domains or protein tags (e.g. NLS, EDLL, VPR), and orange indicates terminator sequences. For actRNA sequences, specific RNA features are highlighted as follows. The tRNA processing element is shown in violet, the tracrRNA sequence in dark green, and the MS2 scaffold in dark blue. The 20-nt guide spacer region is indicated by N characters. All sequences are shown in the 5′-3′ orientation.

Element	Sequence
pNOS::FLuc:tNOS	GATCATGAGCGGAGAATTAAGGGAGTCACGTTATGACCCCCGCCG<br>ATGACGCGGGACAAGCCGTTTTACGTTTGGAACTGACAGAACCGC<br>AACGTTGAAGGAGCCACTCAGCCGCGGGTTTCTGGAGTTTAATGAG<br>CTAAGCACATACGTCAGAAACCATTATTGCGCGTTCAAAAGTCGCC<br>TAAGGTCACTATCAGCTAGCAAATATTTCTTGTCAAAAATGCTCCACT<br>GACGTTCCATAAATTCCCCTCGGTATCCAATTAGAGTCTCATATTCA<br>CTCTCAATCCAAATAATCTGCACCGGATCTTCTGGAAGCACCATGG<br>AAGATGCAAAGAACATTAAGAAAGGGCCTGCACCATTCTATCCACT<br>TGAGGACGGTACAGCCGGTGAGCAATTGCATAAGGCCATGAAGAG<br>ATATGCACTTGTACCAGGGACGATAGCCTTTACTGATGCTCACATTG<br>AGGTCAATATCACTTATGCGGAATATTTTGAAATGTCAGTCAGATTGG<br>CAGAAGCTATGAAAAGGTATGGCTTAAATACCAATCACAGAATTGTG<br>GTGTGTTCTGAGAATAGTTTACAGTTCTTCATGCCAGTGTTGGGTGC<br>ACTATTTATTGGTGTAGCTGTTGCTCCAGCTAACGACATTTACAATGA<br>GAGAGAACTACTCAACTCTATGAATATTTCACAGCCCACAGTCGTAT<br>TTGTGAGCAAGAAAGGATTGCAGAAGATACTTAACGTTCAGAAGAAA<br>CTTCCGATTATACAGAAGATCATTATTATGGACAGCAAAACTGACTA<br>CCAAGGCTTTCAGTCTATGTATACATTTGTGACATCTCATTTACCTCC<br>AGGCTTTAATGAGTATGACTTCGTTCCAGAATCATTTGATCGTGATAA<br>GACTATCGCTCTTATTATGAATAGCTCTGGATCCACTGGTCTGCCGA<br>AGGGAGTAGCATTGCCTCATAGGACAGCTTGTGTTAGGTTTTCACAC<br>GCAAGAGATCCGATATTTGGAAATCAGATCATTCCTGATACGGCTAT<br>ATTGAGTGTTGTACCATTTCACCATGGCTTTGGAATGTTTACCACACT<br>TGGGTACTTGATTTGCGGCTTTAGAGTGGTACTGATGTACAGGTTCG<br>AGGAGGAACTTTTCCTACGAAGTCTCCAAGATTACAAAATCCAATCC<br>GCTTTGTTAGTTCCTACACTGTTTTCCTTCTTCGCGAAATCTACCCTG<br>ATTGACAAATATGATCTCTCTAATCTCCACGAAATAGCAAGTGGTGG<br>TGCACCTCTTTCAAAAGAGGTTGGAGAAGCAGTTGCCAAACGTTTTC<br>ATCTACCAGGTATCAGACAAGGATATGGTCTTACTGAGACAACATCA<br>GCCATATTAATTACCCCTGAAGGCGATGATAAGCCTGGTGCTGTTG<br>GAAAAGTTGTTCCATTCTTCGAAGCAAAAGTGGTAGACTTGGATACT<br>GGCAAGACGCTTGGAGTCAACCAAAGAGGTGAACTGTGCGTTAGA<br>GGGCCTATGATCATGTCTGGGTACGTTAACAATCCTGAGGCAACCA<br>ATGCGTTAATTGACAAAGATGGCTGGTTACATAGTGGAGATATTGCG<br>TACTGGGACGAAGATGAGCACTTCTTCATAGTGGATAGGTTGAAGT<br>CTCTCATCAAGTACAAAGGTTATCAAGTAGCTCCTGCTGAATTAGAG<br>TCAATCCTTCTTCAACATCCCAACATTTTCGATGCTGGAGTGGCCG<br>GATTGCCCGATGATGATGCCGGTGAATTGCCCGCAGCTGTTGTTGT<br>GCTGGAACATGGTAAGACTATGACAGAGAAGGAAATAGTGGACTAT<br>GTCGCTAGCCAAGTTACTACTGCTAAGAAACTCCGTGGAGGGGTTG<br>TCTTTGTTGATGAAGTCCCAAAAGGATTGACTGGGAAACTTGATGCT<br>CGAAAGATAAGAGAGATTCTCATTAAAGCTAAGAAGGGTGGAAAATC

	CAAACTAAAGGGCTAAGACCTAAGCTAGCCTAGAATCGATCGTTCAAACATTTGGCAATAAAGTTTCTTAAGATTGAATCCTGTTGCCGGTCTTGCGATGATTATCATATAATTTCTGTTGAATTACGTTAAGCATGTAATAATTAACATGTAATGCATGACGTTATTTATGAGATGGGTTTTTATGATTAGAGTCCCGCAATTATACATTTAATACGCGATAGAAAACAAAATATAGCGCGCAAACTAGGATAAATTATCGCGCGCGGTGTCATCTATGTTACTAGATC
pNOStrc::Fluc:tNOS	GAACCGCAACGTTGAAGGAGCCACTCAGCCGCGGGTTTCTGGAGTTTAATGAGCTAAGCACATACGTCAGAAACCATTATTGCGCGTTCAAAAGTCGCCTAAGGTCACTATCAGCTAGCAAATATTTCTTGTCAAAAATGCTCCACTGACGTTCCATAAATTCCCCTCGGTATCCAATTATCTGGAAGCACCATGGAAGATGCAAAGAACATTAAGAAAGGGCCTGCACCATTCTATCCACTTGAGGACGGTACAGCCGGTGAGCAATTGCATAAGGCCATGAAGAGATATGCACTTGTACCAGGGACGATAGCCTTTACTGATGCTCACATTGAGGTCAATATCACTTATGCGGAATATTTTGAAATGTCAGTCAGATTGGCAGAAGCTATGAAAAGGTATGGCTTAAATACCAATCACAGAATTGTGGTGTGTTCTGAGAATAGTTTACAGTTCTTCATGCCAGTGTTGGGTGCACTATTTATTGGTGTAGCTGTTGCTCCAGCTAACGACATTTACAATGAGAGAGAACTACTCAACTCTATGAATATTTCACAGCCCACAGTCGTATTTGTGAGCAAGAAAGGATTGCAGAAGATACTTAACGTTCAGAAGAAACTTCCGATTATACAGAAGATCATTATTATGGACAGCAAAACTGACTACCAAGGCTTTCAGTCTATGTATACATTTGTGACATCTCATTTACCTCCAGGCTTTAATGAGTATGACTTCGTTCCAGAATCATTTGATCGTGATAAGACTATCGCTCTTATTATGAATAGCTCTGGATCCACTGGTCTGCCGAAGGGAGTAGCATTGCCTCATAGGACAGCTTGTGTTAGGTTTTCACACGCAAGAGATCCGATATTTGGAAATCAGATCATTCCTGATACGGCTATATTGAGTGTTGTACCATTTCACCATGGCTTTGGAATGTTTACCACACTTGGGTACTTGATTTGCGGCTTTAGAGTGGTACTGATGTACAGGTTCGAGGAGGAACTTTTCCTACGAAGTCTCCAAGATTACAAAATCCAATCCGCTTTGTTAGTTCCTACACTGTTTTCCTTCTTCGCGAAATCTACCCTGATTGACAAATATGATCTCTCTAATCTCCACGAAATAGCAAGTGGTGGTGCACCTCTTTCAAAAGAGGTTGGAGAAGCAGTTGCCAAACGTTTTCATCTACCAGGTATCAGACAAGGATATGGTCTTACTGAGACAACATCAGCCATATTAATTACCCCTGAAGGCGATGATAAGCCTGGTGCTGTTGGAAAAGTTGTTCCATTCTTCGAAGCAAAAGTGGTAGACTTGGATACTGGCAAGACGCTTGGAGTCAACCAAAGAGGTGAACTGTGCGTTAGAGGGCCTATGATCATGTCTGGGTACGTTAACAATCCTGAGGCAACCAATGCGTTAATTGACAAAGATGGCTGGTTACATAGTGGAGATATTGCGTACTGGGACGAAGATGAGCACTTCTTCATAGTGGATAGGTTGAAGTCTCTCATCAAGTACAAAGGTTATCAAGTAGCTCCTGCTGAATTAGAGTCAATCCTTCTTCAACATCCCAACATTTTCGATGCTGGAGTGGCCGGATTGCCCGATGATGATGCCGGTGAATTGCCCGCAGCTGTTGTTGTGCTGGAACATGGTAAGACTATGACAGAGAAGGAAATAGTGGACTATGTCGCTAGCCAAGTTACTACTGCTAAGAAACTCCGTGGAGGGGTTGTCTTTGTTGATGAAGTCCCAAAAGGATTGACTGGAAACTTGATGCTCGAAAGATAAGAGAGATTCTCATTAAAGCTAAGAAGGGTGGAAAATCCAAACTAAAGGGCTAAGACCTAAGCTAGCCTAGAATCGATCGTTCAAACATTTGGCAATAAAGTTTCTTAAGATTGAATCCTGTTGCCGGTCTTGCGATGATTATCATATAATTTCTGTTGAATTACGTTAAGCATGTAATAATTAACATGTAATGCATGACGTTATTTATGAGATGGGTTTTTATGATTAGAGTCCCGCAATTATACATTTAATACGCGATAGAAAAC

	AAAATATAGCGCGCAAACTAGGATAAATTATCGCGCGCGGTGTCAT<br>CTATGTTACTAGATC
35S::RLuc:tNOS	AGATTAGCCTTTTCAATTTCAGAAAGAATGCTAACCCACAGATGGTTA<br>GAGAGGCTTACGCAGCACGACTCATCAAGACGATCTACCCGAGCA<br>ATAATCTCCAGGAAATCAAATACCTTCCCAAGAAGGTTAAAGATGCA<br>GTCAAAAGATTCAGGACTAACTGCATCAAGAACACAGAGAAAGATA<br>TATTTCTCAAGATCAGAAGTACTATTCCAGTATGGACGATTCAAGGCT<br>TGCTTCACAAACCAAGGCAAGTAATAGAGATTGGAGTCTCTAAAAAG<br>GTAGTTCCCACTGAATCAAAGGCCATGGAGTCAAAGATTCAAATAG<br>AGGACCTAACAGAACTCGCCGTAAAGACTGGCGAACAGTTCATAC<br>AGAGTCTCTTACGACTCAATGACAAGAAGAAAATCTTCGTCAACATG<br>GTGGAGCACGACACACTTGTCTACTCCAAAAATATCAAAGATACAG<br>TCTCAGAAGAGCAAAGGGCAATTGAGACTTTTCAACAAAGGGTAATA<br>TCCGGAAACCTCCTCGGATTCCATTGCCCAGCTATCTGTCACTTTAT<br>TGTGAAGATAGTGGAAAAGGAAGGTGGCTCCTACAAATGCCATCAT<br>TGCGATAAAGGAAAGGCCATCGTTGAAGATGCCTCTGCCGACAGT<br>GGTCCCAAAGATGGACCCCCACCCACGAGGAGCATCGTGGAAAA<br>AGAACACGTTCCAACCACGACTTCAAAGCAAGTGGATTGATGTGAT<br>ATCTCCACTGACGTAAGGGATGACGCACAATCCCACTATCCTTCGC<br>AAGACCCTTCCTCTATATAAGGAAGTTCATTTCATTTGGAGAGAACA<br>CGTCTGGAAGCACCATGACGAGTAAAGTGTATGATCCTGAGCAAAG<br>GAAGAGAATGATCACAGGTCCTCAATGGTGGGCTAGGTGTAAACAG<br>ATGAATGTTCTTGACTCTTTCATCAACTACTACGATTCTGAGAAACAT<br>GCCGAAAACGCAGTGATTTTCCTCCATGGAAATGCTGCTTCAAGCT<br>ATCTCTGGAGACACGTTGTCCCACATATAGAACCAGTAGCTAGATG<br>CATTATACCGGATTTGATTGGTATGGGCAAAAGTGGGAAATCAGGG<br>AATGGATCCTACAGGTTATTGGACCACTACAAGTATCTCACAGCTTG<br>GTTTGAACTACTTAACTTGCCTAAGAAGATCATATTCGTGGGACATGA<br>TTGGGGAGCATGTCTTGCCTTTCACTATTCCTATGAACATCAGGACA<br>AAATAAAGGCCATTGTCCATGCAGAGTCTGTTGTTGACGTGATTGAA<br>AGCTGGGATGAATGGCCTGACATTGAGGAGGATATTGCGTTAATCA<br>AGAGTGAAGAAGGCGAGAAAATGGTTCTTGAAAACAATTTCTTTGTC<br>GAAACTATGTTGCCGTCTAAAATCATGAGAAAACTGGAACCAGAAG<br>AATTCGCAGCATACTTAGAACCCTTTAAGGAGAAAGGAGAAGTCAG<br>ACGACCAACTCTGTCTTGGCCTAGAGAGATTCCATTGGTTAAGGGT<br>GGCAAACCAGATGTTGTACAAATTGTGCGAAATTATAACGCTTATCT<br>GCGTGCTTCAGATGATCTTCCTAAGATGTTTATTGAGTCAGATCCCG<br>GTTTCTTTAGCAATGCGATTGTAGAGGGTGCTAAGAAATTTCCAAATA<br>CCGAATTCGTGAAGGTTAAAGGACTACACTTTTCTCAAGAAGATGCA<br>CCTGATGAGATGGGGAAGTACATAAAGTCCTTTGTAGAGCGTGTTCT<br>TAAGAATGAGCAGAAGGGCTAAGACCTAAGCTAGCCTAGAATCGAT<br>CGTTCAAACATTTGGCAATAAAGTTTCTTAAGATTGAATCCTGTTGCC<br>GGTCTTGCGATGATTATCATATAATTTCTGTTGAATTACGTTAAGCATG<br>TAATAATTAACATGTAATGCATGACGTTATTTATGAGATGGGTTTTTATG<br>ATTAGAGTCCCGCAATTATACATTTAATACGCGATAGAAAACAAAATA<br>TAGCGCGCAAACTAGGATAAATTATCGCGCGCGGTGTCATCTATGT<br>TACTAGATC
35S::MCP:VPR:tNOS	AGATTAGCCTTTTCAATTTCAGAAAGAATGCTAACCCACAGATGGTTA<br>GAGAGGCTTACGCAGCACGACTCATCAAGACGATCTACCCGAGCA<br>ATAATCTCCAGGAAATCAAATACCTTCCCAAGAAGGTTAAAGATGCA<br>GTCAAAAGATTCAGGACTAACTGCATCAAGAACACAGAGAAAGATA

	TATTTCTCAAGATCAGAAGTACTATTCCAGTATGGACGATTCAAGGCT<br>TGCTTCACAAACCAAGGCAAGTAATAGAGATTGGAGTCTCTAAAAAG<br>GTAGTTCCCACTGAATCAAAGGCCATGGAGTCAAAGATTCAAATAG<br>AGGACCTAACAGAACTCGCCGTAAAGACTGGCGAACAGTTCATAC<br>AGAGTCTCTTACGACTCAATGACAAGAAGAAAATCTTCGTCAACATG<br>GTGGAGCACGACACACTTGTCTACTCCAAAAATATCAAAGATACAG<br>TCTCAGAAGAGCAAAGGGCAATTGAGACTTTTCAACAAAGGGTAATA<br>TCCGGAAACCTCCTCGGATTCCATTGCCCAGCTATCTGTCACTTTAT<br>TGTGAAGATAGTGGAAAAGGAAGGTGGCTCCTACAAATGCCATCAT<br>TGCGATAAAGGAAAGGCCATCGTTGAAGATGCCTCTGCCGACAGT<br>GGTCCCAAAGATGGACCCCCACCCACGAGGAGCATCGTGGAAAA<br>AGAACACGTTCCAACCACGACTTCAAAGCAAGTGGATTGATGTGAT<br>ATCTCCACTGACGTAAGGGATGACGCACAATCCCACTATCCTTCGC<br>AAGACCCTTCCTCTATATAAGGAAGTTCATTTCATTTGGAGAGAACA<br>CGTCTGGAAGCACCATGATGGCAAGCAACTTCACACAGTTCGTCC<br>TCGTGGACAACGGTGGGACCGGGGACGTGACGGTAGCACCATCC<br>AACTTCGCAAACGGAGTAGCCGAGTGGATAAGCTCAAACTCAAGG<br>TCCCAGGCATACAAAGTCACATGCTCCGTGAGGCAGAGTTCCGCA<br>CAGAACCGCAAATACACGATCAAAGTCGAGGTGCCGAAAGTGGCA<br>ACCCAGACCGTAGGTGGCGTGGAGTTGCCAGTCGCCGCATGGCG<br>GTCATACTTAAACATGGAGCTCACGATCCCGATATTCGCAACAAAC<br>AGCGACTGCGAGTTAATAGTAAAAGCGATGCAGGGTTTACTCAAAG<br>ACGGCAACCCAATCCCAAGTGCAATAGCAGCGAACAGTGGAATAT<br>ACTCAGATCCGAAGAAGAAACGCAAAGTGGACCCTAAGAAGAAAC<br>GAAAAGTTGACCCAAAGAAGAAACGTAAAGTGGATGCCCTCGACG<br>ATTTTGATTTGGATATGTTAGGATCTGACGCGCTGGATGATTTCGATC<br>TGGATATGCTTGGCAGCGATGCTCTCGACGATTTTGATCTTGATATG<br>CTAGGAAGTGATGCACTAGATGATTTCGACCTCGATATGTTGCCAAA<br>GAAGAAGAGAAAGGTCGACCCAAAGAAGAAGCGCAAAGTGCCAA<br>CGCAAGCTGGAGAAGGTACTCTTTCAGAGGCCCTTCTTCAACTACA<br>ATTTGATGATGAGGACCTCGGCGCTCTCCTTGGTAATTCTACTGACC<br>CCGCTGTATTTACTGATTTAGCGTCAGTAGATAATTCAGAGTTTCAAC<br>AATTGCTTAATCAAGGTATTCCAGTTGCTCCACATACTACTGAACCA<br>ATGCTTATGGAGTATCCAGAAGCTATAACACGACTGGTAACGGGCG<br>CCCAACGTCCACCAGACCCTGCACCAGCACCACTAGGTGCTCCT<br>GGACTACCGAACGGCTTGCTAAGTGGGGATGAAGATTTCTCTTCCA<br>TTGCTGACATGGACTTTTCTGCACTTCTAATAAATAGTCGGTCAAGTG<br>GGTCCAGGGACTCTAGAGAGGGTATGTTCTTACCAAAACCAGAAG<br>CAGGTTCTGCCATCTCAGATGTTTTCGAAGGACGAGAAGTTTGTCAA<br>CCTAAGCGTATAAGACCTTTCCACCCACCTGGTAGCCCTTGGGCTA<br>ATAGGCCGCTTCCGGCTTCTCTGGCCCCGACTCCTACTGGGCCC<br>GTGCACGAACCCGTGGGATCTCTAACTCCTGCTCCTGTACCGCAA<br>CCTTTGGACCCTGCACCTGCTGTTACCCCGGAAGCATCCCATTTG<br>CTTGAAGATCCTGACGAGGAGACTAGTCAAGCAGTGAAGGCTCTG<br>CGTGAAATGGCTGACACCGTAATACCTCAGAAAGAGGAAGCAGCT<br>ATTTGCGGGCAGATGGATCTGTCTCACCCACCACCGAGAGGTCAC<br>TTAGACGAATTGACTACTACCCTCGAATCAATGACAGAGGACCTAA<br>ATTTAGATTCCCCTTTGACGCCTGAGCTTAATGAAATATTAGACACGT<br>TTCTAAATGATGAATGTTTACTGCACGCTATGCACATATCTACGGGGT<br>TGTCTATTTTCGATACGAGTTTGTTCAAGGGCTAAGACCTAAGCTAG<br>CCTAGAATCGATCGTTCAAACATTTGGCAATAAAGTTTCTTAAGATTG

	AATCCTGTTGCCGGTCTTGCGATGATTATCATATAATTTCTGTTGAATT<br>ACGTTAAGCATGTAATAATTAACATGTAATGCATGACGTTATTTATGAG<br>ATGGGTTTTTATGATTAGAGTCCCGCAATTATACATTTAATACGCGAT<br>AGAAAACAAAATATAGCGCGCAAACTAGGATAAATTATCGCGCGCG<br>GTGTCATCTATGTTACTAGATC
35S::NLS:dSpCas9:EDLL:tNOS	AGATTAGCCTTTTCAATTTCAGAAAGAATGCTAACCCACAGATGGTTA<br>GAGAGGCTTACGCAGCACGACTCATCAAGACGATCTACCCGAGCA<br>ATAATCTCCAGGAAATCAAATACCTTCCCAAGAAGGTTAAAGATGCA<br>GTCAAAAGATTCAGGACTAACTGCATCAAGAACACAGAGAAAGATA<br>TATTTCTCAAGATCAGAAGTACTATTCCAGTATGGACGATTCAAGGCT<br>TGCTTCACAAACCAAGGCAAGTAATAGAGATTGGAGTCTCTAAAAAG<br>GTAGTTCCCACTGAATCAAAGGCCATGGAGTCAAAGATTCAAATAG<br>AGGACCTAACAGAACTCGCCGTAAAGACTGGCGAACAGTTCATAC<br>AGAGTCTCTTACGACTCAATGACAAGAAGAAAATCTTCGTCAACATG<br>GTGGAGCACGACACACTTGTCTACTCCAAAAATATCAAAGATACAG<br>TCTCAGAAGAGCAAAGGGCAATTGAGACTTTTCAACAAAGGGTAATA<br>TCCGGAAACCTCCTCGGATTCCATTGCCCAGCTATCTGTCACTTTAT<br>TGTGAAGATAGTGGAAAAGGAAGGTGGCTCCTACAAATGCCATCAT<br>TGCGATAAAGGAAAGGCCATCGTTGAAGATGCCTCTGCCGACAGT<br>GGTCCCAAAGATGGACCCCCACCCACGAGGAGCATCGTGGAAAA<br>AGAACACGTTCCAACCACGACTTCAAAGCAAGTGGATTGATGTGAT<br>ATCTCCACTGACGTAAGGGATGACGCACAATCCCACTATCCTTCGC<br>AAGACCCTTCCTCTATATAAGGAAGTTCATTTCATTTGGAGAGAACA<br>CGTCTGAACAATGCTGCAGCCTAAGAAGAAGAGAAAGGTTGGAGG<br>AGTCGACTCGAGTGCGGCCGCCACCATGGACAAGAAGTACTCCAT<br>TGGGCTCGAAATCGGCACAAACAGCGTCGGCTGGGCCGTCATTA<br>CGGACGAGTACAAGGTGCCGAGCAAAAAATTCAAAGTTCTGGGCA<br>ATACCGATCGCCACAGCATAAAGAAGAACCTCATTGGCGCCCTCC<br>TGTTCGACTCCGGGGAGACGGCCGAAGCCACGCGGCTCAAAAGA<br>ACAGCACGGCGCAGATATACCCGCAGAAAGAATCGGATCTGCTAC<br>CTGCAGGAGATCTTTAGTAATGAGATGGCTAAGGTGGATGACTCTTT<br>CTTCCATAGGCTGGAGGAGTCCTTTTTGGTGGAGGAGGATAAAAAG<br>CACGAGCGCCACCCAATCTTTGGCAATATCGTGGACGAGGTGGC<br>GTACCATGAAAAGTACCCAACCATATATCATCTGAGGAAGAAGCTT<br>GTAGACAGTACTGATAAGGCTGACTTGCGGTTGATCTATCTCGCGC<br>TGGCGCATATGATCAAATTTCGGGGACACTTCCTCATCGAGGGGGA<br>CCTGAACCCAGACAACAGCGATGTCGACAAACTCTTTATCCAACTG<br>GTTCAGACTTACAATCAGCTTTTCGAAGAGAACCCGATCAACGCAT<br>CCGGAGTTGACGCCAAAGCAATCCTGAGCGCTAGGCTGTCCAAAT<br>CCCGGCGGCTCGAAAACCTCATCGCACAGCTCCCTGGGGAGAAG<br>AAGAACGGCCTGTTTGGTAATCTTATCGCCCTGTCACTCGGGCTGA<br>CCCCCAACTTTAAATCTAACTTCGACCTGGCCGAAGATGCCAAGCT<br>TCAACTGAGCAAAGACACCTACGATGATGATCTCGACAATCTGCTG<br>GCCCAGATCGGCGACCAGTACGCAGACCTTTTTTTGGCGGCAAAG<br>AACCTGTCAGACGCCATTCTGCTGAGTGATATTCTGCGAGTGAACA<br>CGGAGATCACCAAAGCTCCGCTGAGCGCTAGTATGATCAAGCGCT<br>ATGATGAGCACCACCAAGACTTGACTTTGCTGAAGGCCCTTGTCAG<br>ACAGCAACTGCCTGAGAAGTACAAGGAAATTTTCTTCGATCAGTCTA<br>AAAATGGCTACGCCGGATACATTGACGGCGGAGCAAGCCAGGAG<br>GAATTTTACAAATTTATTAAGCCCATCTTGGAAAAAATGGACGGCAC<br>CGAGGAGCTGCTGGTAAAGCTTAACAGAGAAGATCTGTTGCGCAAA

	CAGCGCACTTTCGACAATGGAAGCATCCCCCACCAGATTCACCTG<br>GGCGAACTGCACGCTATCCTCAGGCGGCAAGAGGATTTCTACCCC<br>TTTTTGAAAGATAACAGGGAAAAGATTGAGAAAATCCTCACATTTCGG<br>ATACCCTACTATGTAGGCCCCCTCGCCCGGGGAAATTCCAGATTC<br>GCGTGGATGACTCGCAAATCAGAAGAGACTATCACTCCCTGGAAC<br>TTCGAGGAAGTCGTGGATAAGGGGGCCTCTGCCCAGTCCTTCATC<br>GAAAGGATGACTAACTTTGATAAAAATCTGCCTAACGAAAAGGTGCT<br>TCCTAAACACTCTCTGCTGTACGAGTACTTCACAGTTTATAACGAGC<br>TCACCAAGGTCAAATACGTCACAGAAGGGATGAGAAAGCCAGCAT<br>TCCTGTCTGGAGAGCAGAAGAAAGCTATCGTGGACCTCCTCTTCAA<br>GACGAACCGGAAAGTTACCGTGAAACAGCTCAAAGAAGATTATTTC<br>AAAAAGATTGAATGTTTCGACTCTGTTGAAATCAGCGGAGTGGAGGA<br>TCGCTTCAACGCATCCCTGGGAACGTATCACGATCTCCTGAAAATC<br>ATTAAAGACAAGGACTTCCTGGACAATGAGGAGAACGAGGACATTC<br>TTGAGGACATTGTCCTCACCCTTACGTTGTTTGAAGATAGGGAGATG<br>ATTGAAGAACGCTTGAAAACTTACGCTCATCTCTTCGACGACAAAGT<br>CATGAAACAGCTCAAGAGGCGCCGATATACAGGATGGGGGCGGC<br>TGTCAAGAAAACTGATCAATGGGATCCGAGACAAGCAGAGTGGAAA<br>GACAATCCTGGATTTTCTTAAGTCCGATGGATTTGCCAACCGGAACT<br>TCATGCAGTTGATCCATGATGACTCTCTCACCTTTAAGGAGGACATC<br>CAGAAAGCACAAGTTTCTGGCCAGGGGGACAGTCTCCACGAGCA<br>CATCGCTAATCTTGCAGGTAGCCCAGCTATCAAAAAGGGAATACTG<br>CAGACCGTTAAGGTCGTGGATGAACTCGTCAAAGTAATGGGAAGG<br>CATAAGCCCGAGAATATCGTTATCGAGATGGCCCGAGAGAACCAA<br>ACTACCCAGAAGGGACAGAAGAACAGTAGGGAAAGGATGAAGAG<br>GATTGAAGAGGGTATAAAAGAACTGGGGTCCCAAATCCTTAAGGAA<br>CACCCAGTTGAAAACACCCAGCTTCAGAATGAGAAGCTCTACCTGT<br>ACTACCTGCAGAACGGCAGGGACATGTACGTGGATCAGGAACTGG<br>ACATCAATCGGCTCTCCGACTACGACGTGGATGCTATCGTGCCCC<br>AGTCTTTTCTCAAAGATGATTCTATTGATAATAAAGTGTTGACAAGATC<br>CGATAAAAATAGAGGGAAGAGTGATAACGTCCCCTCAGAAGAAGTT<br>GTCAAGAAAATGAAAAATTATTGGCGGCAGCTGCTGAACGCCAAAC<br>TGATCACACAACGGAAGTTCGATAATCTGACTAAGGCTGAACGAGG<br>TGGCCTGTCTGAGTTGGATAAAGCCGGCTTCATCAAAAGGCAGCTT<br>GTTGAGACACGCCAGATCACCAAGCACGTGGCCCAAATTCTCGAT<br>TCACGCATGAACACCAAGTACGATGAAAATGACAAACTGATTCGAG<br>AGGTGAAAGTTATTACTCTGAAGTCTAAGCTGGTTTCAGATTTCAGAA<br>AGGACTTTCAGTTTTATAAGGTGAGAGAGATCAACAATTACCACCAT<br>GCGCATGATGCCTACCTGAATGCAGTGGTAGGCACTGCACTTATCA<br>AAAAATATCCCAAGCTTGAATCTGAATTTGTTTACGGAGACTATAAAG<br>TGTACGATGTTAGGAAAATGATCGCAAAGTCTGAGCAGGAAATAGG<br>CAAGGCCACCGCTAAGTACTTCTTTTACAGCAATATTATGAATTTTTT<br>CAAGACCGAGATTACACTGGCCAATGGAGAGATTCGGAAGCGACC<br>ACTTATCGAAACAAACGGAGAAACAGGAGAAATCGTGTGGGACAA<br>GGGTAGGGATTTCGCGACAGTCCGGAAGGTCCTGTCCATGCCGC<br>AGGTGAACATCGTTAAAAAGACCGAAGTACAGACCGGAGGCTTCT<br>CCAAGGAAAGTATCCTCCCGAAAAGGAACAGCGACAAGCTGATCG<br>CACGCAAAAAAGATTGGGACCCCAAGAAATACGGCGGATTCGATT<br>CTCCTACAGTCGCTTACAGTGTACTGGTTGTGGCCAAAGTGGAGAA<br>AGGGAAGTCTAAAAAACTCAAAAGCGTCAAGGAACTGCTGGGCAT<br>CACAATCATGGAGCGATCAAGCTTCGAAAAAAACCCCATCGACTTT

	CTCGAGGCGAAAGGATATAAAGAGGTCAAAAAAGACCTCATCATTA<br>AGCTTCCCAAGTACTCTCTCTTTGAGCTTGAAAACGGCCGGAAACG<br>AATGCTCGCTAGTGCGGGCGAGCTGCAGAAAGGTAACGAGCTGG<br>CACTGCCCTCTAAATACGTTAATTTCTTGTATCTGGCCAGCCACTAT<br>GAAAAGCTCAAAGGATCTCCCGAAGATAATGAGCAGAAGCAGCTG<br>TTCGTGGAACAACACAAACACTACCTTGATGAGATCATCGAGCAAA<br>TAAGCGAATTCTCCAAAAGAGTGATCCTCGCCGACGCTAACCTCGA<br>TAAGGTGCTTTCTGCTTACAATAAGCACAGGGATAAGCCCATCAGG<br>GAGCAGGCAGAAAACATTATCCACTTGTTTACTCTGACCAACTTGG<br>GCGCGCCTGCAGCCTTCAAGTACTTCGACACCACCATAGACAGAA<br>AGCGGTACACCTCTACAAAGGAGGTCCTGGACGCCACACTGATTC<br>ATCAGTCAATTACGGGGCTCTATGAAACAAGAATCGACCTCTCTCA<br>GCTCGGTGGAGACAGCAGGGCTGACCCCAAGAAGAAGAGGAAG<br>GTGAAGGGAGATCCAAAGAAGAAAAGAAAAGTTGATCCCAAGAAGA<br>AACGCAAAGTTGACCCTAAGAAGAAACGAAAAGTTGAGGTATTCGA<br>GTTCGAGTACTTAGACGACAAGGTTCTTGAGGAGTTGTTAGACTCC<br>GAGGAGAGGAAGAGGTAAAATCGATCGTTCAAACATTTGGCAATAA<br>AGTTTCTTAAGATTGAATCCTGTTGCCGGTCTTGCGATGATTATCATA<br>TAATTTCTGTTGAATTACGTTAAGCATGTAATAATTAACATGTAATGCAT<br>GACGTTATTTATGAGATGGGTTTTTATGATTAGAGTCCCGCAATTATA<br>CATTTAATACGCGATAGAAAACAAAATATAGCGCGCAAACTAGGATA<br>AATTATCGCGCGCGGTGTCATCTATGTTACTAGATC
35S::NLS:dSaCas9:EDLL:tNOS	AGATTAGCCTTTTCAATTTCAGAAAGAATGCTAACCCACAGATGGTTA<br>GAGAGGCTTACGCAGCACGACTCATCAAGACGATCTACCCGAGCA<br>ATAATCTCCAGGAAATCAAATACCTTCCCAAGAAGGTTAAAGATGCA<br>GTCAAAAGATTCAGGACTAACTGCATCAAGAACACAGAGAAAGATA<br>TATTTCTCAAGATCAGAAGTACTATTCCAGTATGGACGATTCAAGGCT<br>TGCTTCACAAACCAAGGCAAGTAATAGAGATTGGAGTCTCTAAAAAG<br>GTAGTTCCCACTGAATCAAAGGCCATGGAGTCAAAGATTCAAATAG<br>AGGACCTAACAGAACTCGCCGTAAAGACTGGCGAACAGTTCATAC<br>AGAGTCTCTTACGACTCAATGACAAGAAGAAAATCTTCGTCAACATG<br>GTGGAGCACGACACACTTGTCTACTCCAAAAATATCAAAGATACAG<br>TCTCAGAAGAGCAAAGGGCAATTGAGACTTTTCAACAAAGGGTAATA<br>TCCGGAAACCTCCTCGGATTCCATTGCCCAGCTATCTGTCACTTTAT<br>TGTGAAGATAGTGGAAAAGGAAGGTGGCTCCTACAAATGCCATCAT<br>TGCGATAAAGGAAAGGCCATCGTTGAAGATGCCTCTGCCGACAGT<br>GGTCCCAAAGATGGACCCCCACCCACGAGGAGCATCGTGGAAAA<br>AGAACACGTTCCAACCACGACTTCAAAGCAAGTGGATTGATGTGAT<br>ATCTCCACTGACGTAAGGGATGACGCACAATCCCACTATCCTTCGC<br>AAGACCCTTCCTCTATATAAGGAAGTTCATTTCATTTGGAGAGAACA<br>CGTCTGAACAATGCTGCAGCCTAAGAAGAAGAGAAAGGTTGGAGG<br>AGTCGACTCGAGTGCGGCCGCCACCATGAAGAGGAACTACATCCT<br>CGGACTCGCTATCGGAATCACCTCTGTGGGATACGGAATCATCGAT<br>TACGAGACTAGGGATGTGATCGATGCTGGTGTGAGACTCTTCAAAG<br>AGGCTAACGTTGAGAACAACGAGGGAAGAAGGTCTAAGAGGGGAG<br>CTAGAAGGCTCAAGAGAAGAAGAAGGCACAGAATCCAGAGGGTGA<br>AGAAGCTCCTCTTCGATTACAACCTCCTCACCGATCACTCTGAGCT<br>TTCTGGAATCAACCCTTACGAGGCAAGAGTGAAGGGACTCTCTCAG<br>AAGTTGTCTGAGGAAGAGTTCTCTGCTGCTTTGCTCCACCTTGCTAA<br>GAGAAGGGGTGTGCATAACGTGAACGAGGTGGAAGAGGATACCG<br>GAAACGAGCTTTCTACCAAAGAGCAGATCTCTAGGAACTCTAAGGC

	TCTCGAAGAGAAGTACGTGGCAGAGCTTCAGCTTGAGAGACTCAA<br>GAAAGATGGTGAGGTGAGGGGATCTATCAACAGGTTCAAGACCTCT<br>GATTACGTGAAAGAAGCTAAGCAGCTCCTCAAGGTGCAGAAGGCTT<br>ACCATCAGCTCGATCAGTCTTTCATCGATACCTACATCGATCTTCTC<br>GAGACTAGAAGGACCTACTACGAGGGACCTGGTGAAGGATCTCCA<br>TTCGGATGGAAGGATATCAAAGAATGGTACGAGATGCTCATGGGAC<br>ACTGCACTTACTTCCCTGAAGAACTCAGATCTGTGAAGTACGCTTAC<br>AACGCTGATCTCTACAACGCTCTTAACGATCTCAACAACCTCGTGA<br>TCACCAGGGATGAGAACGAGAAGCTTGAGTACTACGAGAAGTTCC<br>AGATCATCGAGAACGTGTTCAAGCAAAAGAAGAAGCCTACCCTCAA<br>GCAGATCGCTAAAGAGATCCTTGTTAACGAGGAAGATATCAAGGGA<br>TACAGGGTGACCTCTACCGGAAAGCCTGAGTTCACTAACCTCAAG<br>GTTTACCACGATATTAAGGATATTACCGCTAGAAAAGAGATTATTGAG<br>AACGCTGAGCTTCTCGATCAAATCGCTAAGATCCTTACCATCTACC<br>AGTCATCTGAGGATATCCAAGAGGAATTGACCAACCTCAACTCAGA<br>GCTTACCCAAGAAGAGATCGAGCAGATCAGTAACTTGAAGGGTTAC<br>ACCGGAACCCACAACCTCTCACTCAAGGCTATCAACCTCATCCTC<br>GATGAGCTTTGGCACACCAACGATAACCAGATCGCAATCTTCAACA<br>GACTCAAGCTCGTGCCTAAGAAAGTGGATCTCTCTCAGCAGAAAGA<br>GATTCCTACCACCCTCGTGGATGATTTCATCCTCTCACCTGTTGTGA<br>AGAGATCTTTCATCCAGTCTATCAAGGTGATCAACGCTATTATCAAG<br>AAATACGGACTCCCTAACGATATCATCATCGAGTTGGCTAGGGAAA<br>AGAACTCAAAGGATGCTCAAAAGATGATCAACGAGATGCAGAAGAG<br>AAACAGGCAGACCAACGAGAGGATCGAAGAGATCATCAGGACCA<br>CCGGAAAAGAGAACGCTAAGTACCTCATTGAGAAGATCAAGCTCC<br>ACGATATGCAAGAGGGAAAGTGCCTCTACTCTCTCGAGGCTATCC<br>CTCTTGAGGATCTCCTTAACAACCCTTTCAACTACGAAGTGGATGC<br>CATCATCCCTAGATCTGTGTCTTTCGATAACTCTTTCAACAACAAGGT<br>GCTCGTGAAGCAAGAGGAAAACTCTAAGAAGGGTAACAGGACCCC<br>TTTCCAGTACCTCTCTTCATCTGATTCTAAGATCTCATACGAGACTTT<br>CAAGAAGCACATCCTCAACCTCGCTAAGGGAAAGGGTAGGATCTC<br>TAAGACAAAGAAAGAGTACCTTCTCGAGGAAAGGGATATCAACAGA<br>TTCTCTGTTCAGAAGGATTTCATCAACAGAAACCTTGTGGATACCAG<br>GTACGCTACCAGGGGTCTTATGAACCTTCTCAGGTCTTACTTCAGG<br>GTGAACAACCTTGATGTGAAGGTGAAGTCAATCAACGGTGGATTCA<br>CCTCATTCCTTAGAAGAAAATGGAAGTTTAAGAAAGAGAGGAACAAG<br>GGTTATAAGCACCACGCTGAGGATGCTCTCATTATCGCTAACGCAG<br>ATTTCATTTTCAAAGAGTGGAAGAAGTTGGATAAGGCTAAAAAGGTG<br>ATGGAAAACCAGATGTTCGAGGAAAAGCAGGCTGAGTCTATGCCTG<br>AGATCGAGACTGAGCAAGAGTACAAAGAAATCTTCATCACCCCTCA<br>CCAGATCAAGCACATCAAAGATTTCAAGGATTACAAGTACTCTCACA<br>GAGTGGATAAGAAGCCAAACAGAGAGCTTATCAACGATACCCTCTA<br>CTCAACCAGAAAGGATGATAAGGGAAACACCCTCATCGTGAACAAT<br>CTCAACGGACTCTACGATAAGGATAACGATAAGCTCAAGAAGCTCA<br>TTAACAAGTCTCCAGAGAAGTTGCTCATGTACCACCACGATCCTCA<br>GACCTACCAAAAGCTCAAGCTCATCATGGAACAGTACGGTGATGA<br>GAAGAACCCTCTCTACAAGTATTACGAGGAAACCGGTAACTACCTC<br>ACCAAGTACTCAAAAAAGGATAATGGACCTGTGATCAAGAAGATTAA<br>GTACTACGGAAACAAGCTCAACGCACACCTCGATATCACCGATGAT<br>TACCCTAACAGTAGAAACAAGGTGGTGAAGCTTTCACTCAAGCCTT<br>ACAGGTTCGATGTGTACCTCGATAACGGTGTGTACAAGTTCGTGAC

	CGTGAAGAACCTCGATGTTATCAAAAAAGAAAACTACTATGAGGTTA<br>ACTCTAAGTGCTACGAGGAAGCTAAGAAGTTGAAGAAGATCTCTAA<br>CCAGGCAGAGTTCATTGCTTCATTCTACAACAACGATCTTATCAAGA<br>TTAACGGTGAGCTTTACAGGGTTATCGGAGTGAACAACGATTTGCTC<br>AACAGGATCGAGGTGAACATGATTGATATCACCTACAGGGAATACC<br>TCGAGAATATGAACGATAAGAGGCCTCCTAGAATCATCAAGACTAT<br>CGCTTCTAAGACCCAGAGTATCAAGAAGTACTCTACCGATATCCTC<br>GGAAACCTCTACGAGGTTAAGTCAAAGAAGCACCCTCAGATCATTA<br>AGAAGGGATCAAGGGCTGATCCTAAGAAGAAGAGGAAGGTTAAGG<br>GAGATCCAAAGAAGAAAAGAAAAGTTGATCCCAAGAAGAAACGCAA<br>AGTTGACCCTAAGAAGAAACGAAAAGTTGAGGTATTCGAGTTCGAGT<br>ACTTAGACGACAAGGTTCTTGAGGAGTTGTTAGACTCCGAGGAGAG<br>GAAGAGGTAAAATCGATCGTTCAAACATTTGGCAATAAAGTTTCTTAA<br>GATTGAATCCTGTTGCCGGTCTTGCGATGATTATCATATAATTTCTGTT<br>GAATTACGTTAAGCATGTAATAATTAACATGTAATGCATGACGTTATTT<br>ATGAGATGGGTTTTTATGATTAGAGTCCCGCAATTATACATTTAATAC<br>GCGATAGAAAACAAAATATAGCGCGCAAACTAGGATAAATTATCGC<br>GCGCGGTGTCATCTATGTTACTAGATC
35S::p19:tNOS	AGATTAGCCTTTTCAATTTCAGAAAGAATGCTAACCCACAGATGGTTA<br>GAGAGGCTTACGCAGCACGACTCATCAAGACGATCTACCCGAGCA<br>ATAATCTCCAGGAAATCAAATACCTTCCCAAGAAGGTTAAAGATGCA<br>GTCAAAAGATTCAGGACTAACTGCATCAAGAACACAGAGAAAGATA<br>TATTTCTCAAGATCAGAAGTACTATTCCAGTATGGACGATTCAAGGCT<br>TGCTTCACAAACCAAGGCAAGTAATAGAGATTGGAGTCTCTAAAAAG<br>GTAGTTCCCACTGAATCAAAGGCCATGGAGTCAAAGATTCAAATAG<br>AGGACCTAACAGAACTCGCCGTAAAGACTGGCGAACAGTTCATAC<br>AGAGTCTCTTACGACTCAATGACAAGAAGAAAATCTTCGTCAACATG<br>GTGGAGCACGACACACTTGTCTACTCCAAAAATATCAAAGATACAG<br>TCTCAGAAGAGCAAAGGGCAATTGAGACTTTTCAACAAAGGGTAATA<br>TCCGGAAACCTCCTCGGATTCCATTGCCCAGCTATCTGTCACTTTAT<br>TGTGAAGATAGTGGAAAAGGAAGGTGGCTCCTACAAATGCCATCAT<br>TGCGATAAAGGAAAGGCCATCGTTGAAGATGCCTCTGCCGACAGT<br>GGTCCCAAAGATGGACCCCCACCCACGAGGAGCATCGTGGAAAA<br>AGAACACGTTCCAACCACGACTTCAAAGCAAGTGGATTGATGTGAT<br>ATCTCCACTGACGTAAGGGATGACGCACAATCCCACTATCCTTCGC<br>AAGACCCTTCCTCTATATAAGGAAGTTCATTTCATTTGGAGAGAACA<br>CGTCTGGAAGCACCATGGAACGAGCTATACAAGGAAACGACGCTA<br>GGGAACAAGCTAACAGTGAACGTTGGGATGGAGGATCAGGAGGTA<br>CCACTTCTCCCTTCAAACTTCCTGACGAAAGTCCGAGTTGGACTGA<br>GTGGCGGCTACATAACGATGAGACGAATTCGAATCAAGATAATCCC<br>CTTGGTTTCAAGGAAAGCTGGGGTTTCGGGAAAGTTGTATTTAAGAG<br>ATATCTCAGATACGACAGGACGGAAGCTTCACTGCACAGAGTCCTT<br>GGATCTTGGACGGGAGATTCGGTTAACTATGCAGCATCTCGATTTTT<br>CGGTTTCGACCAGATCGGATGTACCTATAGTATTCGGTTTCGAGGA<br>GTTAGTATCACCGTTTCTGGAGGGTCGCGAACTCTTCAGCATCTCT<br>GTGAGATGGCAATTCGGTCTAAGCAAGAACTGCTACAGCTTGCCC<br>CAATCGAAGTGGAAAGTAATGTATCAAGAGGATGCCCTGAAGGTAC<br>TGAAACCTTCGAAAAAGAAAGCGAGTAAAAGGGCTAAGACCTAAGC<br>TAGCCTAGAATCGATCGTTCAAACATTTGGCAATAAAGTTTCTTAAGA<br>TTGAATCCTGTTGCCGGTCTTGCGATGATTATCATATAATTTCTGTTGA<br>ATTACGTTAAGCATGTAATAATTAACATGTAATGCATGACGTTATTTAT

	GAGATGGGTTTTTATGATTAGAGTCCCGCAATTATACATTTAATACGC<br>GATAGAAAACAAAATATAGCGCGCAAACTAGGATAAATTATCGCGC<br>GCGGTGTCATCTATGTTACTAGATC
pU6::actRNA$_{SpCas9}$<br><br>tRNA, 20 nt spacer, tracrRNA and MS2 scaffold	CTTTTTTTCTTCTTCTTCGTTCATACAGTTTTTTTTTGTTTATCAGCTTAC<br>ATTTTCTTGAACCGTAGCTTTCGTTTTCTTCTTTTTAACTTTCCATTCGG<br>AGTTTTTGTATCTTGTTTCATAGTTTGTCCCAGGATTAGAATGATTAGG<br>CATCGAACCTTCAAGAATTTGATTGAATAAAACATCTTCATTCTTAAGA<br>TATGAAGATAATCTTCAAAAGGCCCCTGGGAATCTGAAAGAAGAGA<br>AGCAGGCCCATTTATATGGGAAAGAACAATAGTATTTCTTATATAGGC<br>CCATTTAAGTTGAAAACAATCTTCAAAAGTCCCACATCGCTTAGATAA<br>GAAAACGAAGCTGAGTTTATATACAGCTAGAGTCGAAGTAGTG<br><br>NNNNNNNNNNN<br>NNNNNNNNN<br>GGGA<br><br>CCTTTTTT
pU6::actRNA$_{SaCas9}$<br>tRNA, 20 nt spacer, tracrRNA and MS2 scaffold	CTTTTTTTCTTCTTCTTCGTTCATACAGTTTTTTTTTGTTTATCAGCTTAC<br>ATTTTCTTGAACCGTAGCTTTCGTTTTCTTCTTTTTAACTTTCCATTCGG<br>AGTTTTTGTATCTTGTTTCATAGTTTGTCCCAGGATTAGAATGATTAGG<br>CATCGAACCTTCAAGAATTTGATTGAATAAAACATCTTCATTCTTAAGA<br>TATGAAGATAATCTTCAAAAGGCCCCTGGGAATCTGAAAGAAGAGA<br>AGCAGGCCCATTTATATGGGAAAGAACAATAGTATTTCTTATATAGGC<br>CCATTTAAGTTGAAAACAATCTTCAAAAGTCCCACATCGCTTAGATAA<br>GAAAACGAAGCTGAGTTTATATACAGCTAGAGTCGAAGTAGTG<br><br>NNNNNNNNNNN<br>NNNNNNNNN<br>GGGA<br><br>CCTTTTTT

Supplementary Table S2: Used actRNAs. Given are the used Sequence of the gRNA motif of the specific actRNAs and which Cas9 ortholog was specific for the given actRNA.

actRNA Name	Cas9	Sequence gRNA	Comment
actRAN1$_{Sp,pNOS}$	*Sp*Cas9	GGGAATTTATGGAACGTCAG	
actRAN2$_{Sp,pNOS}$	*Sp*Cas9	GAGACTCTAATTGGATACCG	
actRAN3$_{Sp,pNOS}$	*Sp*Cas9	GCGCGTTCAAAAGTCGCCTA	
actRAN5$_{Sp,pNOS}$	*Sp*Cas9	TGCTAGCTGATAGTGACCTT	
actRAN6$_{Sp,pNOS}$	*Sp*Cas9	ATGACCCCCGCCGATGACGC	
actRAN9$_{Sp,pNOS}$	*Sp*Cas9	TGACAGAACCGCAACGTTGA	
actRAN12$_{Sp,pNOS}$	*Sp*Cas9	GCCACTCAGCCGCGGGTTTC	Same target sequence as actRNA1$_{Sa,pNOS}$
actRNA1$_{Sa,pNOS}$	*Sa*Cas9	GCCACTCAGCCGCGGGTTTC	Same target sequence as actRAN12$_{Sp,pNOS}$

actRNA2$_{\mathrm{Sa},\mathit{pNOS}}$	*Sa*Cas9	AATTCCCCTCGGTATCCAAT	
actRNA3$_{\mathrm{Sa},\mathit{pNOS}}$	*Sa*Cas9	GACTCTAATTGGATACCGAG	
actRNA4$_{\mathrm{Sa},\mathit{pNOS}}$	*Sa*Cas9	GTTGAAGGAGCCACTCAGCC	
actRNA6$_{\mathrm{Sa},\mathit{pNOS}}$	*Sa*Cas9	GCTTGTCCCGCGTCATCGGC	
actRNA7$_{\mathrm{Sa},\mathit{pNOS}}$	*Sa*Cas9	GATCATGAGCGGAGAATTAA	
actRNA7$_{\mathrm{Sa},\mathit{pNOS}}$	*Sa*Cas9	CTCAATCCAAATAATCTGCA	

Supplementary Table S3. PAM-compatible target site analysis across endogenous tomato promoters for different Cas9 variants. The table summarises the number of potential CRISPR target sites within promoter regions of selected endogenous tomato genes based on Sol Genomics Network database involved in the phenylalanine pathway, stratified by Cas9 ortholog and PAM recognition sequence analysed by chopchop.cbu.uib.no. Canonical, PAM-altered, and PAM-relaxed Cas9 variants are shown, including *Sp*Cas9 (NGG), *Sp*Cas9 VQR (NGA), *Sp*Cas9 EQR (NGAG), *Sp*G (NGN), *Sa*Cas9 (NNGRRT), *Sa*Cas9 KKH (NNNRRT), and *Sth1*Cas9 (NNAGAAW). For each promoter-Cas9 combination, counts are reported as follows. (**a**), total number of PAM-compatible gRNA target sites identified within the promoter region; (**b**), number of gRNAs with zero predicted off-target sites allowing ≤ 2 mismatches; (**c**), number of gRNAs with zero predicted off-target sites ≤ 1 mismatch. This analysis was used to assess the practical impact of PAM constraints and off-target stringency on endogenous targetability in plant CRISPRa applications.

	SpCas9			SpCas9 VQR			SpCas9 EQR			SpG			SaCas9			SaCas9 KKH			Sth1Cas9		
	NGG			NGA			NGAG			NGN			NNGRRT			NNNRRT			NNAGAAW		
	a	b	c	a	b	c	a	b	c	a	b	c	a	b	c	a	b	c	a	b	c
**Phenylalanine ammonia-lyase**																					
**Solyc09g007920**	27	11	8	36	9	17	12	5	3	94	10	49	13	9	2	53	13	23	5	4	0
**Solyc09g007900**	14	7	7	33	11	19	1	1	0	85	20	51	4	4	0	48	15	31	5	5	0
**Solyc09g007910**	22	10	6	21	7	7	6	3	0	68	15	28	7	3	2	46	16	20	1	1	0
**Solyc09g007890**	16	5	9	27	21	3	7	2	1	71	10	46	6	5	1	77	11	12	6	5	1
**Solyc10g086180**	51	29	19	41	22	16	9	6	2	99	36	55	18	12	6	59	31	27	2	2	0
**Solyc05g056170**	25	3	7	12	7	5	2	2	0	60	5	29	9	4	4	42	9	28	5	4	0
**Caffeoyl-CoA O-methyltransferase**																					
**Solyc02g093270**	7	7	0	29	12	14	8	4	4	63	24	35	7	6	1	37	19	16	4	3	0
**Solyc02g093250**	21	0	2	30	1	1	4	0	0	87	0	4	7	0	0	62	0	2	3	0	0
**Solyc02g093230**	15	9	1	21	8	3	3	1	0	57	15	18	8	3	0	46	10	4	2	0	0
**Solyc01g107910**	20	13	7	33	16	14	4	2	2	86	33	39	6	5	0	46	17	19	4	3	1
**Solyc10g050160**	27	26	1	43	36	7	7	7	0	113	62	48	15	15	0	55	36	17	5	5	0
**Solyc04g063210**	4	1	0	42	7	9	15	2	7	93	14	31	7	5	2	39	9	16	4	2	0
**Solyc03g032220**	7	3	3	25	9	13	0	0	0	47	4	29	5	3	1	40	10	20	7	6	1
**Solyc09g082660**	18	12	5	24	17	6	5	5	0	82	33	46	6	5	1	47	22	19	0	0	0
**4-coumarate CoA ligase**																					

**Solyc06g068650**	32	13	8	49	24	11	12	9	1	123	32	45	13	7	1	52	23	16	4	4	0
**Solyc03g117870**	27	16	7	29	15	10	8	8	0	85	20	48	10	8	2	45	18	21	4	4	0
**Solyc12g042460**	6	0	5	28	6	14	10	5	2	49	6	26	6	3	2	48	12	23	3	1	1
**Solyc07g008360**	13	0	1	20	3	1	3	1	0	81	7	8	2	0	1	37	3	1	2	1	0
